# An Insight into miR-1290: An Oncogenic miRNA with Diagnostic Potential

**DOI:** 10.3390/ijms23031234

**Published:** 2022-01-22

**Authors:** Małgorzata Guz, Witold Jeleniewicz, Marek Cybulski

**Affiliations:** Department of Biochemistry and Molecular Biology, Medical University of Lublin, 20-093 Lublin, Poland; witoldjeleniewicz@umlub.pl (W.J.); marekcybulski@umlub.pl (M.C.)

**Keywords:** miR-1290, oncomir, cancer, prognosis, biomarker, diagnosis, miRNA

## Abstract

For more than two decades, the view of the roles of non-coding RNAs (ncRNAs) has been radically changing. These RNA molecules that are transcribed from our genome do not have the capacity to encode proteins, but are critical regulators of gene expression at different levels. Our knowledge is constantly enriched by new reports revealing the role of these new molecular players in the development of many pathological conditions, including cancer. One of the ncRNA classes includes short RNA molecules called microRNAs (miRNAs), which are involved in the post-transcriptional control of gene expression affecting various cellular processes. The aberrant expression of miRNAs with oncogenic and tumor-suppressive function is associated with cancer initiation, promotion, malignant transformation, progression and metastasis. Oncogenic miRNAs, also known as oncomirs, mediate the downregulation of tumor-suppressor genes and their expression is upregulated in cancer. Nowadays, miRNAs show promising application in diagnosis, prediction, disease monitoring and therapy response. Our review presents a current view of the oncogenic role of miR-1290 with emphasis on its properties as a cancer biomarker in clinical medicine.

## 1. Introduction

Cancer incidence and mortality is growing rapidly worldwide. Important factors that facilitate this phenomenon are aging, growth of the global population, and changes in the prevalence and distribution of the main risk factors that are tightly associated with social and economic development [1]. Statisticians predicted about 2 million new cancer cases (1,896,160) worldwide and approximately 600 thousand cancer deaths (608,570) in the United States in 2021 [2]. The key concepts recognized as molecular and cellular mechanisms underlying cancer development are: (1) the ability of cancer cells to sustain proliferative signaling, (2) evading growth suppressors, (3) resisting cell death, (4) inducing angiogenesis, (5) activating invasion and metastasis, (6) enabling replicative immortality, (7) deregulating cellular energetics, (8) avoiding immune destruction, (9) genome instability and mutations, and (10) tumor-promoting inflammation [3,4,5]. Moreover, Senga and Grose, in their work published in 2021, proposed four novel hallmarks of cancer: dedifferentiation and transdifferentiation, altered microbiome, altered neuronal signaling, and epigenetic alterations that complete the characteristics of heterogenous pathologies such as neoplastic diseases [6]. Each cell in a multicellular organism contains the same DNA; however, epigenetic information regulates how our genome is read [7]. This means that the combination of genes that are expressed or repressed determines cellular morphology and functions [8]. Epigenetic changes including DNA methylation, histone acetylation and methylation, post-translational modifications and non-coding RNAs contribute to tumorigenesis regardless of the DNA sequence [9]. Non-coding RNAs related to epigenetic regulation are: long-non-coding RNAs (lncRNAs), PIWI-interacting RNAs (piRNAs), small interfering RNAs (siRNAs), and microRNAs (miRNAs) that are directly involved in cancerogenesis or indirectly participate in this process by the regulation of other epigenetic events [10].

## 2. Overview of miRNAs’ Biogenesis and Function

In the canonical biosynthesis pathway, most primary miRNAs transcripts (pri-miRNA) are synthesized by polymerase II in association with transcription factors and epigenetic regulators from its own promoter or the promoter that is shared with a host gene [11]. Pri-miRNAs, which have a hairpin structure ranging in length from hundreds to thousands of base pairs (bp), are processed in two steps by two endoribonucleases, Drosha and Dicer [12]. In the nucleus, Drosha, along with its co-factor protein called DiGeorge Syndrome Critical Region 8 (DGCR8), forms a microprocessor complex that processes pri-miRNA to hairpin intermediates called precursor miRNA (pre-miRNA) with a length of ~70 bp [13]. Pre-miRNA are exported to the cytoplasm by an exportin 5 XPO5 and GTP-binding nuclear protein Ran-GTP, where they are processed by Dicer and double-stranded RNA-binding protein by cutting the terminal loop, which generates ~22-nucleotide mature duplexes [14]. The 5p strand of the pre-miRNA hairpin structure arises from the 5′-end while the 3p strand is derived from 3′-end [15]. The silencing of gene expression is carried out by the RNA-induced silencing complex (RISC), which is assembled through a few steps, during which the miRNA duplex is loaded into one of the proteins from the Argonaute family (AGO 1–4 in humans) with the aid of ATP, and Hsp70/Hsp90 machinery [16]. Next, one of the strands of the duplex, known as the passenger strand, is generally degraded by AGO’s slice activity, but it depends on the cell type or cellular environment [14]. MiRNAs target 3′ untranslated regions (UTRs) of even thousands of messenger RNAs (mRNAs), but miRNAs are also able to interact with mRNA 5′UTRs and coding sequences [14]. The number of miRNA-binding sites within regulated target mRNAs influences the efficiency of the regulation of gene expression, the same as the distance between miRNA-binding sites, because if more target sites are present on one transcript, then the silencing efficiency is higher, probably because of cooperative interactions between miRISCs that are in the near neighborhood [17]. The degree of complementarity between miRNA seed region (position 2–8 of the mature miRNAs) and miRNA recognition elements (MRE) in the target mRNA determine its fate, which leads to AGO2-dependent slicing or the inhibition of translation and mRNA decay [18]. Fully complementary targets are cleaved by AGO2, but generally in animals a partial complementarity between miRNA and mRNA dominates [19]. The inhibition of translation occurs after the recruitment of RISC-bound miRNA to mRNA by interfering with the eucaryotic initiation factor 4F complex (eIF4F) [19]. However, it is possible to translationally reactivate mRNAs repressed by miRNAs. The repression of translation triggers the deadenylation and degradation of target mRNA [20]. For this purpose, trinucleotide repeat-containing 6 proteins (TNRC6A, -B, -C) are required as a partners for AGO proteins and they are involved in miRNA–mRNA repression in mammalian cells, inducing a two-phase deadenylation process that recruits PAN2-PAN3 and CCR4:NOT complexes [17,21]. This process is followed by the removal of 5′-M^7^-guanosyl-diphosphate of mRNAs catalyzed by decapping enzyme DCP2 and its cofactor DCP1A. Decapping is also enhanced by stimulatory factors: EDC3, EDC4, Pat1b and DEAD-box helicase 6 (DDX6) [22]. At the end, mRNAs are rapidly degraded by 5′-exonuclease XRN1 [23].

MiRNAs are recognized as a master regulators of gene expression that are implicated in a wide range of physiological and pathological processes, including tumorigenesis [24]. Their aberrant expression in tumors reflects their functions, because overexpressed miRNAs act as oncogenes that target tumor-suppressor genes (TSG), which promotes malignant transformation [25]. On the other hand, miRNAs, which are tumor suppressors, are downregulated in cancer cells and oncogenes are their molecular targets [26]. Genetic mutations and epigenetic alterations are responsible for the deregulated expression of miRNAs, and the same follows in the altered tumor microenvironment, where secreted growth factors or cytokines may affect the expression of these small RNAs [27]. Other causes of aberrant miRNA expression in cancer are the aberrant activity of transcription factors and mutations in the components of miRNA processing machinery [28,29].

In addition to the canonical biosynthesis pathway of miRNA, the alternative biogenesis of miRNAs is Drosha- or Dicer-independent [30]. The main routes bypassing the Drosha pathway generate: (m7G)-capped pre-miRNAs that are transported to the cytoplasm by exportin 1 without cleavage by Drosha, or miRNAs such as “mirtrons” processed by the spliceosome and debranching enzymes from introns [31,32]. Some human mirtrons are splicing independent and are called mirtron-like or “simtrons” [33]. These pre-miRNAs are considered as Drosha-dependent small RNAs, because their biogenesis is unaffected by the deletion of DGCR8, Dicer, or EXPO5 AGO2, but reduced after Drosha knockout [34]. An example of the Dicer-independent biosynthesis of miRNA is the maturation of pre-miR-451 that is too short for Dicer cleavage and requires processing by AGO2, the activity of which is augmented by eukaryotic translation initiation factor (EIF1A) [35,36]. On the other hand, Dicer and DGCR8 are required for the synthesis of snoRNA-derived miRNAs, where the latter can degrade snoRNA independently of Drosha and thus has an impact on the stability of this alternative miRNAs [37]. Short hairpin-derivative miRNAs, such as miR-320 and miR-484, are Dicer dependent and DGCR8 independent because they lack a microprocessor-binding sequence [38]. Additionally, small RNAs that are released from tRNA (tsRNAs) are crucial for numerous physiological and pathological processes such as gene expression, translation initiation and elongation, intergenerational inheritance, stress granule assembly, ribosome biogenesis and apoptosis [39,40]. These small RNAs, similar in size to miRNAs, are divided according to their length into: stress-induced tRNAs (tiRNAs) and shorter tRNA-derived fragments (tRFs) derived from mature or precursor tRNAs [40,41].

## 3. Circulating miRNAs as Promising Non-Invasive Markers

**Figure 1:**
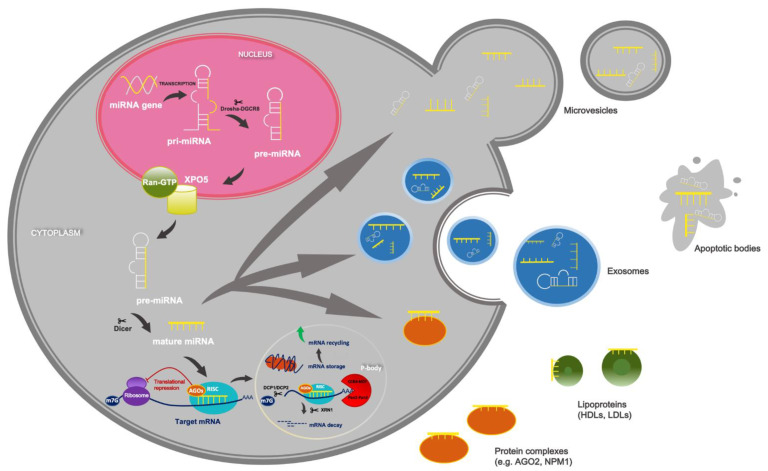
Biogenesis of miRNAs and their extracellular forms.

Most miRNAs are located within cells, but some of them are released and present in different biofluids including plasma, serum, saliva, urine, tears, cerebrospinal fluid, amniotic fluid, pancreatic juice, bile, gastric juice, synovial fluid, and breast milk [42]. Therefore, it is considered that miRNAs play an important role in the communication between cells, and they are defined as circulating miRNAs. Recent findings suggest that cells in the neighborhood of or located distantly from cells releasing miRNAs are instructed by circulating miRNAs, because miRNAs are transmitted as a “message” that leads to physiological or pathological effects in recipient cells, resulting in the progression of disease [43]. In the case of neoplastic diseases, it is confirmed that miRNAs secreted from the primary site of the tumor promote proliferation, invasion, angiogenesis, distant metastasis, and remodeling of the tumor microenvironment [44]. Outside the cell, miRNAs exist in membrane vesicles such as exosomes, microvesicles, and apoptotic bodies [45]. Additionally, they may be associated with lipoproteins, particularly high-density lipoproteins (HDLs) and with intermediate lipoproteins (IDLs) or low-density lipoproteins (LDLs). They can also be a part of complexes with proteins such as AGO2 and nucleophosmin 1 (NPM1) [46] (Figure 1).

MiRNAs appearing in these forms in extracellular spaces are stable, because they are not degraded by endogenous RNases [33]. Furthermore, circulating miRNAs are resistant to different stressing conditions, such as long-term storage, repeated freeze–thaw cycles, boiling, and variations in pH [47,48]. Since the discovery of circulating miRNAs, the significance of these molecular players has increased, particularly as potential non-invasive biomarkers. The possibility of the collection of miRNAs in a non-invasive or minimally invasive manner prevents many invasive medical procedures such as tissue biopsy (e.g., kidney or liver biopsy, bone marrow procedures) [48]. Another limitation of traditional biopsy is the small amount of diagnostic material depending on the size of needle or areas of sampling [49]. In addition, intratumor heterogeneity should also be considered as tumor cells divide, accumulate additional mutations, and gain new properties, for example apoptosis resistance and invasiveness [50]. In addition, epigenetic alterations are the cause of differences between the phenotypes of some cancer cell populations within a tumor, which also contributes to tumor heterogeneity [50]. In theory, any part of the tumor may shed secretory vesicles, proteins, nucleic acids or cells into the bloodstream, which confirms that the cancer-derived miRNAs present in biofluids may be a more representative form of sampling in the future than traditional biopsy [49]. An essential feature of ideal biological markers, except non-invasiveness, is their detection at an early stage of disease, specificity, and sensitivity. In the context of circulating miRNAs, many studies confirmed the altered expression of plasma or serum miRNAs before the obvious clinical symptoms appeared and diagnosis was clearly confirmed by biopsy or imaging techniques [51]. For example, upregulated levels of miR-21, miR-20a, miR-141, miR-145, miR-155 and miR-223 were noted in the plasma of patients with non-small cell lung cancer (NSCLC) at stages I and II [51]. Fan et al. identified miR-16, miR-21, miR-155 and miR-195 in early breast cancer patients in comparison with healthy individuals [52]. Other miRNAs were mentioned as markers for the early detection and discrimination of bladder cancer from non-cancer and other types of bladder tumor (miR-6087, miR-6724-5p, miR-3960, miR-1343-5p, miR-1185-1-3p, miR-6831-5p and miR-4695-5p) [53]. Some miRNAs were considered as novel markers in the early diagnosis of esophageal squamous dysplasia (ESD), the precancerous lesion of esophageal squamous cell carcinoma (ESCC), where miR-16-5p, miR-197-5p, miR-451a, and miR-92a-3p distinguished patients with ESCC from healthy individuals, and miR-16-5p, miR-320c, miR-638, and miR-92a-3p distinguished ESD from healthy controls. These two miRNA panels when taken together had a better diagnostic performance in the differentiation of ESD and ESCC from healthy patients than carcinoembryonic antigen (CAE) and carbohydrate antigen 19-9 (CA 19-9) [54]. Additionally, different subtypes of cancers may be distinguished by the evaluation of circulating miRNAs expression. Approximately 75% of breast cancers are positive for estrogen receptor (ER) and/or progesterone receptor (PR), where ER-positive tumors express ER, PR, ER-responsive elements or other genes that encode proteins of luminal epithelial cells, and therefore were termed the luminal group [55]. The differential expression of miR-373 was revealed in triple-negative breast cancer (TNBC) patients in comparison with luminal carcinomas, and its expression was higher in (ER)-/(PR)- subtypes than in receptor-positive breast cancer [56]. In lung cancer patients, levels of miR-16-5p and 486-5p were elevated in two subclasses of NSCLC, adenocarcinoma (ADC) and squamous cell carcinoma (SCC), in comparison with healthy controls, but the stable expression of miR-9 was noted in healthy individuals and the overall group of NSCLC patients, with a significant decline only in the ADC group [51].

Despite advanced knowledge in research focused on the functions of non-coding RNAs, the use of miRNAs as diagnostic biomarkers is limited [57]. It is well documented that the quality of pre-analytical and analytical methods for the detection of miRNAs is extremely important [58]. Sample collection, storage, transportation, repeating thawing and freezing, aliquoting, and the selection of centrifugation conditions during samples preparation (type of rotor, centrifugation force, time and temperature) are crucial, because they may alter the levels of miRNAs derived from biofluids [47]. Some studies showed the differential expression of circulating miRNAs in arterial blood compared with venous blood in pathological conditions, which may indicate that the selection of blood sampling method is key, but is not the only variable in the detection of circulating miRNAs [59,60]. In addition, many studies are not consistent in assessing the levels of specific miRNAs in serum and plasma where miRNA expression patterns are without differences; miRNA levels are higher in plasma than in serum; or their levels are higher in serum than in plasma, where hypothetically these small RNAs are released from platelets during coagulation [61,62,63,64]. The crucial step during the preparation of plasma and serum samples involves the removal of the cellular components of blood (red blood cells, white blood cells and platelets) to limit the release of miRNAs derived from these cells and therefore avoid the RNA contamination of samples and false-positive results during miRNAs detection [58]. In the case of plasma, the choice of anticoagulant (EDTA, citrate or heparin) used for miRNAs isolation is critical for the quality of miRNAs profiling due to the impact of anticoagulants on the results of quantitative reverse-transcription PCR (qRT-PCR) [47,65]. Moreover, reducing the hemolysis of samples is also mandatory, because the expression of some miRNAs may be changed in hemolyzed specimens [58]. Therefore, the relative expression of, for example, erythrocyte-specific miR-451 and stable miR-23a may be used as an indicator of hemolysis [66].

Other variables affecting the profiling of circulating miRNAs include their extraction methods due to the low concentration of these small RNAs in blood, the forms of their transport, and contaminants present in blood, for example, heme [47]. Nowadays, different methods are used for the detection of miRNAs in samples such as qRT-PCR, miRNA microarrays, and next-generation sequencing (NGS); however, the choice of the proper method depends on the study design. Furthermore, the normalization strategy used for miRNAs quantification is essential, because it affects data interpretation [58,67].

In the search for miRNAs that can be used as diagnostic biomarkers, individual factors influencing the diverse expression of miRNAs, such as single-nucleotide polymorphisms (SNPs), RNA editing and the production of miRNA isoforms (isomirs) should also be considered [68]. Moreover, circulating miRNA levels are related to patients’ age, gender and race [69,70,71,72]. Additionally, factors related to lifestyle such as medication, nutrition and physical activity modulate the profile of circulating miRNAs [47]. Medications such as angiotensin-converting enzyme inhibitors and angiotensin receptor blockers downregulate some miRNAs in patients with coronary artery disease [73]. In patients with knee osteoarthritis, treatment with celecoxib, as one of the most frequently used selective cyclooxygenase-2 (COX-2) inhibitors, influenced the levels of circulating miRNAs before and after therapy [74]. Furthermore, the intake of specific nutrients such as sodium and vitamins D and E is related to circulating miRNA profiles in healthy subjects. Similarly, a diet rich in polyphenols (resveratrol, ellagitannins, ellagic acid, epigallocatechin-3-gallate, genistein, curcumin, diindolylomethane) that maintains human health by tumor-protective and cancer-suppressive effects, and the high consumption of dietary fatty acids are associated with metabolic diseases via the regulation of miRNA expression [75,76,77]. In addition to dietary habits, the type of physical activity is significant, since it is known that long-term exercise alters plasma miRNAs levels in healthy young women. Moreover, the dynamics of miRNAs expression in plasma and urine is changed in full-marathon participants [78,79]. Recently, it was suggested that cigarette smoking, alcohol abuse and the use opioid of drugs such as heroin influence miRNA levels as well [80,81,82,83,84].

All of the above-mentioned methodological considerations and patient-related factors should be taken into account during study design, because the reliable analysis of circulating miRNA profiles will allow their use as biomarkers suitable for clinical medicine.

## 4. Bioinformatics Analysis of miR-1290 Target Genes and Their Functional Annotations

In 2008, the precursors of human miR-1290 were discovered in embryonic stem cells in introns of aldehyde dehydrogenase 4 member A1 gene (*ALDH4A1*) [85]. We used the miRWalk 3.0 database (mirwalk.umm.uni-heidelberg.de, accessed on January 7 2022) [86] to predict target genes (TGs) for miR-1290 and searched only for interactions common with the miRDB database (mirdb.org) [87,88]. A total of 318 genes (Supplementary Materials, Table S1) with the highest probability of interaction (miRWalk score > 0.9) and present in both databases were chosen for Gene Oncology (GO) and Kyoto Encyclopedia of Genes and Genomes (KEGG) functional analysis. The Database for Annotation, Visualization and Integrated Discovery (DAVID, 2021 Update, david-d.ncifcrf.gov, accessed on January 7 2022) [89,90] was used to identify GO categories (biological process, cellular component, and molecular function) and KEGG pathways (Supplementary Materials, Table S2). In the cellular component category, miR-1290 TGs were enriched, especially in nucleoplasm, cytoplasm and nucleus. The most significant molecular functions of miR-1290 TGs were related to protein binding, protein kinase activity and metal ion binding. Among many biological process terms, we discovered that miR-1290 TGs are involved in MAPK cascade, axon guidance and the positive regulation of protein localization to the plasma membrane. The analysis of KEGG pathways revealed that putative miR-1290 targets, among others, are responsible for the regulation of the MAPK signaling pathway, the calcium signaling pathway and gap junctions.

## 5. Physiological Role of miR-1290

Besides its unquestionable role in carcinogenesis, miR-1290 also takes part in several physiological processes, such as embryonic development and the differentiation of cells. Kim et al. suggested that miR-1290 is important for embryo implantation and survival, and that its expression is negatively regulated by estrogens [91]. It was demonstrated that miR-1290 expression significantly increases in bovine endometrium between days 3 and 7 of the estrous cycle, a critical period for implantation [92]. Moreover, exosomal miR-1290 excreted by placental trophoblast cells induces the epithelial–mesenchymal transition of endometrial cells and their migration through targeting LIM/homeobox protein (LHX6), which may confirm its role in successful implantation [93]. Additionally, placental miR-1290 expression is negatively correlated with gestational age and birthweight, which may indicate its role in fetal growth at an early gestation period [94]. MiR-1290 appeared to be essential during the differentiation of neural stem cells into neurons and its diminished expression in early brain development is associated with some pathologies such as autism spectrum disorder [95,96]. The role of miR-1290 in the development of the central nervous system was reflected by its lower expression in the plasma of pregnant women with fetal Down syndrome compared with women with normal pregnancies [97]. Moreover, miR-1290 is engaged in the differentiation of myoblasts and its serum level in patients with muscle atrophy is lower than in healthy individuals [98]. MiR-1290 also induces phenotypic changes from cardiac fibroblasts to endothelial cells, subsequently enhancing angiogenesis and alleviating cardiac injury in the case of myocardial infarction [99]. Similarly, the protective role of miR-1290 on neurons deprived of oxygen and glucose in ischemia was explained by a reduction in apoptosis in the presence of endothelial cells releasing extracellular vesicles containing miR-1290 [100]. Furthermore, elevated miR-1290 was found in exosomes excreted by bone mesenchymal stem cells (MSCs) during their differentiation into chondrocytes, which may play an important role in cartilage regeneration [101]. Cui et al. have shown that miR-1290 belongs to the set of miRNAs increased during hepatic differentiation [102]; however, their further study indicated that its presence is not necessary for the conversion of MSCs into hepatocyte-like cells [103]. MiR-1290 is upregulated during the induction of umbilical cord MSCs into neuron-like cells, which is a promising discovery for the therapy of neurodegenerative disorders [104]. MiR-1290 may also be engaged in ovarian follicle development as it was found in follicular fluid in both microvesicles and vesicle-free fractions [105].

## 6. The Role of miR-1290 in Non-Neoplastic Diseases

It was discovered that miR-1290 participates in some non-neoplastic pathological processes such as inflammatory and infectious diseases, and polycystic ovary syndrome. The analysis of plasma content from patients with heat stroke compared with healthy volunteers revealed elevated levels of exosomes with dysregulated miRNAs related to increased expression of pro-inflammatory and coagulation cascade-activating mRNAs. Among these altered miRNAs, miR-1290 was one of the most upregulated [106]. MiR-1290 was also shown to be increased in the sera of hyperlipidemia patients and it may be engaged in atherosclerosis-related inflammation through targeting GSK-3β in endothelial cells [107]. An in vitro model of non-alcoholic fatty liver disease (NAFLD) revealed that chronic exposure to free fatty acids may induce the intracellular expression of miR-1290 [108] and miR-1290 was elevated in the serum of NAFLD patients compared with healthy controls [109]. MiR-1290 was overexpressed in intestinal specimens from children with necrotizing enterocolitis [110] and an increased plasma level of miR-1290 in premature infants with necrotizing enterocolitis allowed differentiation from neonatal sepsis cases and healthy children [111]. Furthermore, miR-1290 was elevated in peripheral blood dendritic cells from chronic rhinosinusitis patients with nasal polyps [112] and in the duodenal mucosa of coeliac patients [113]. MiR-1290 expression was also higher in plasma from patients with pulmonary fibrosis and it was able to directly target Napsin A proteinase to modulate cell proliferation and TGF-β1-induced fibrosis [114]. On the other hand, miR-1290 expression was decreased in tissue samples from patients with oral submucous fibrosis compared with normal mucosa [115]. The study of Adyshev et al. suggested that miR-1290 may reduce lung inflammation through targeting nonmuscle myosin light-chain kinase that decreases lung endothelium permeability [116]. Additionally, bioinformatics analysis identified miR-1290 in the set of miRNAs downregulated in serum from sepsis patients [117]. Moreover, it was revealed that the sponging of miR-1290 by hsa_circ_0056558 is involved in the regulation of CDK6 to suppress cell proliferation and differentiation while enhancing apoptosis in ankylosing spondylitis [118]. For that reason, the role of miR-1290 during inflammation and in autoimmune disorders needs to be further clarified.

There are also a few reports indicating that miR-1290 is engaged in the pathogenesis of infections with influenza virus [119], HIV-1 [120] or *Mycobacterium leprae* [121].

Serum miR-1290 was found to be elevated in women with polycystic ovary syndrome, a common endocrine disorder in premenopausal women, compared with controls, and it was positively correlated with high levels of testosterone and androstenedione [122].

## 7. Upregulation of miR-1290 Is Associated with Different Types of Cancers

**Figure 2:**
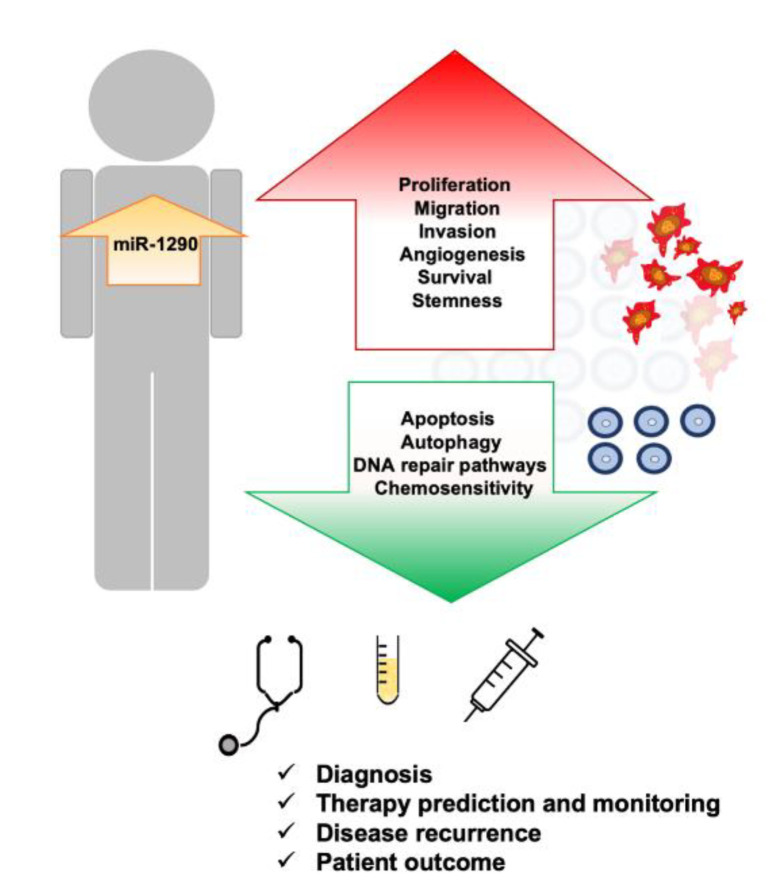
A summary of oncogenic role of miR-1290.

MiR-1290 was detected in various types of malignancies, including colon, pancreas, lung and liver cancer; therefore, its role as an oncomir is well documented. The expression profiles of miRNAs are tumor and tissue specific, and so several miRNAs, among them miR-1290, have a significant role in the development and progression of these pathologies (Figure 2).

In our review, we summarize the role of miR-1290 in different types of tumors, with particular emphasis on its role as a circulating biomarker (Supplementary Materials, Table S3). Moreover, we highlight the clinical significance of miR-1290 as a biomarker in different human neoplasms (Table 1).

### 7.1. Gastrointestinal Cancers

#### 7.1.1. Colorectal Cancer

Colorectal cancer (CRC) is third in terms of incidence and second in terms of mortality worldwide. It is the most common type of cancer in men, after lung and prostate cancers, and the second most common type of cancer in women after breast cancer [139]. CRC is a disease of developed countries, because the main factors that impact tumor development are obesity, sedentary lifestyle, tobacco smoking, and the consumption of red meat and alcohol [140]. Other risk factors are related to inflammatory bowel diseases, diabetes mellitus, cholecystectomy, and hereditary syndromes, such as hereditary non-polyposis colorectal cancer, and adenomatous (familial adenomatous polyposis and MYUTH-associated polyposis) and hamartomatous polyposis syndromes (Peutz–Jaghers syndrome, juvenile polyposis syndrome, and PTEN hamartoma tumors syndrome) [141]. Only a small percentage of CRC cases are a result of hereditary syndromes, whereas most CRC cases are sporadic, accounting for about 95% [142]. The most frequent premalignant precursor lesions of almost all sporadic CRCs are adenomas [143]. Crucial molecular changes in CRC pathogenesis are seen in the chromosomal instability pathway (CIN), which is characterized by aneuploidy and loss of heterozygosity (LOH), microsatellite instability (MSI) caused by a defective DNA mismatch repair (MMR) system, DNA methylation pathway (CpG methylator phenotype pathway), and inflammatory pathway [144]. Additionally, miRNAs are also involved in CRC development [144]. Ma et al. showed that miR-1290 is upregulated in human CRC cells and tissues [145]. It was discovered that a molecular downstream target for miR-1290 is inositol polyphosphatase-4 phosphatase type II (INPP4B), a member of the PI3K/Akt signaling pathway which plays a key role in the initiation and progression of the tumor [146]. MiR-1290 directly binds to 3′-UTR regions of INPP4B that exhibit a dual role in cancer cells [145]. INPP4B functions as a tumor suppressor in prostate, thyroid or bladder cancer, but as an oncomir in CRC cells where it regulates Akt and GSK3 pathways and downregulates tumor-suppressor PTEN, so its role depends on the cellular context and requires further investigation [145,147,148,149]. Other studies also showed that miR-1290 inhibits the expression of tumor-suppressor cyclin-dependent kinase inhibitor 27 (p27) and promotes the expression of cyclin D1, which regulates the transition from the G1 to S phase of the cell cycle and enhances the proliferation of CRC cells [145]. Additionally, the upregulation of miR-1290 in CRC tissues caused the formation of multinucleated cells; miR-1290 activates cell pro-survival pathways such as Akt and NF-κB pathways to maintain cell proliferation in vitro and in vivo [150]. MiR-1290 promotes the reprogramming of cancer cells by targeting kinesin family member 13B (KIF13B), which is involved in cytokinesis, the final step in cell division, that leads to nuclear re-fusion in cells, and aneuploidy, which is present in malignant tumors, including CRC [150]. Moreover, the upregulation of miR-1290 activates the Wnt pathway, which plays a central role in the development of CRC, and further increases c-Myc and Nanog expression [150].

CRC can be classified based on the expression patterns of MMR proteins [151]. MMR genes encode proteins required for the repair of DNA sequence mismatch, correction of base mismatches, and small deletions and insertions [152]. Core members of the MMR system are human mutS homolog 2 (hMSH2) and human mutL homolog 1 (hMLH1); others are hMSH3, hMSH6, hMLH3, and hPMS2 [153]. Patients with a deficient MMR system due to the inactivation of these genes weakly respond or do not respond to 5-fluorouracil-based (5-FU) adjuvant therapy, the first-line therapy applied after surgical resection of CRC in stage II and III [123]. MiR-1290 is upregulated in deficient MMR CRC tissues and 5-FU-resistant cells, and high expression of miR-1290 is a predictor of poor prognosis in patients with stage II and III CRC who receive 5-FU-based chemotherapy. The inhibition of miR-1290 expression in vitro and in vivo enhances sensitivity to 5-FU by targeting hMSH2 [123]. Another study showed that sorafenib, which inhibits the mitogen-activated protein kinase (MAPK) pathway, shows anti-proliferative and anti-angiogenic effects by inhibiting vascular endothelial growth factor receptors (VEGFR). Treatment with sorafenib enhances miR-1290 expression in Caco-2 cells, which may suggest that miR-1290 has as role as a potential indicator of response to this therapy [154].

At least 25–50% of patients with CRC develop metastases to the liver [155]. Comparative analysis of differentially expressed miRNAs between primary CRC and liver metastases showed the upregulation of nine miRNAs, including miR-1290 [156]. Moreover, bioinformatics analysis revealed potential miR-1290 target genes, for example: *AXL*, a gene associated with tumor cell growth, metastasis, invasion, epithelial-to-mesenchymal transition (EMT), angiogenesis, drug resistance and stem cell maintenance; Casp-8 and FADD-like apoptosis regulator (*CFLAR*) encoding an important regulatory protein in the extrinsic apoptotic pathway; growth arrest-specific 7 (*GAS7*), responsible for maintaining microtubule stability and polarization, or thioredoxin-related transmembrane protein 4 (*TMX4*), acting as an endoplasmic reductase required in protein folding and degradation pathways [156,157,158,159,160].

Biopsy has a central role in cancer diagnosis, because it permits clinicians to diagnose disease, helps to choose appropriate treatment, and determines the overall prognosis. The liquid biopsy refers to genetic tests performed on samples extracted from biofluids, in particular from whole blood [161]. Recently, the identification and evaluation of miRNAs that are released from cancer cells into the circulation provided a valuable strategy for diagnosis, even at the early stages of the disease. Usually, CRC patients are diagnosed with advanced stages of the disease, but the evaluation of serum miR-1290 is a promising tool that may help to differentiate adenoma and CRC from healthy controls [124]. Shi et al. demonstrated the high diagnostic value of serum exosomal miR-1290 to differentiate patients at stage I CRC from healthy individuals [125]. Additionally, the high expression of miR-1290 in serum was found to be an independent prognostic marker for predicting poorer overall survival (OS) in CRC patients and was an independent predictor of tumor recurrence after surgery [126].

#### 7.1.2. Pancreatic Cancer

Pancreatic cancer is the 7th leading cause of cancer death in both sexes, which accounts for almost as many deaths as cases in 2020 because of its poor prognosis (deaths: 466,000; cases: 496,000) [1]. The most frequent pancreatic cancer is pancreatic ductal adenocarcinoma (PDAC). The development of pancreatic cancer represents the adenoma–carcinoma sequence: starting from pancreatic intraepithelial neoplasia (PanINs: PanIN-1A, PanIN-1B, II and III) and ending with invasive neoplastic lesions [162]. PDAC may also evolve from other types of precursor lesions, such as intraductal papillary mucinous neoplasms (IPMN) and mucinous cystic neoplasm (MCN) [163]. The disease is considered as familial when two or more first-degree relatives have been previously diagnosed with PDAC, and such patients have a nine times higher risk of developing PDAC [164]. The definition of hereditary PDAC describes this condition as a genetic syndrome with identifiable gene mutation [165]. In this group, PDAC is associated with specific syndromes for which the predisposing genes were identified, including hereditary breast and ovarian cancer (associated with genes: *BRCA1* and *BRCA2*), familial breast cancer (*PALB2* and *ATM*), Peutz–Jaghers syndrome (*STK11*), hereditary pancreatitis (*RRSS1*), familial atypical mole and multiple melanoma (*CDKN2A*), Lynch syndrome or hereditary non-polyposis CRC (MMR genes: *MLH1*, *MSH2*, *MHS6*, *PMS2*), and cystic fibrosis (*CFTR*) [162,166]. Environmental risk factors for PDAC are: cigarette smoking, alcohol consumption, obesity, chronic pancreatitis, and diabetes mellitus [162]. In the context of PDAC, in vivo and in vitro studies confirmed that miR-1290 functions as a promoter of PDAC by targeting 3′-UTR of *IKK1* mRNA and increases the aggressiveness of PDAC [167]. It is known that in pancreas IKK1 protein is a part of the IKK complex involved in the NF-κB signaling pathway, the major pathway connecting inflammation and cancer [168]. Moreover, the upregulation of miR-1290 increases the invasiveness and migration of PDAC cells in vitro and promotes cell proliferation in vitro and in vivo [167]. It is consistent with earlier studies that show that the downregulation of *IKK1* promotes tumor initiation and enhances its progression [169]. Additionally, a meta-analysis showed that high levels of miR-1290 in the blood of patients with PDAC are associated with poorer OS, which confirms its prognostic value [170]. In addition, miR-1290 has a higher expression in older and male PDAC patients undergoing surgical resection [171]. Moreover, the combined detection of circulating miR-1290 along with cancer antigen 19-9 (CA 19-9) may improve the diagnostic accuracy of PDAC [129]. Interestingly, exosomes containing miR-1290, which were isolated from the human pancreatic cell lines, were taken up by neighboring normal pancreatic stellate cells and induced genes involved in fibrogenesis, a process that creates a tumor-faciliatory environment, and stimulates tumor growth and metastasis [172].

#### 7.1.3. Gastric Cancer

Gastric cancer (GC) is a result of a combination of environmental factors and mutations in various genes [173]. The majority of GCs are adenocarcinomas that originate from the epithelial cells of gastric mucosa and the glands, whereas other types arise from the lymphoid tissue and muscles of the stomach [174]. The most frequently used classification of gastric adenocarcinomas are Lauren’s criteria, based on GC histologic subtypes [175] that include two major types: intestinal and diffuse [176]. In 2010 the World Health Organization recognized additional histologic subtypes of gastric tumors, such as: papillary, mucinous, tubular, poorly cohesive, including signet ring cell carcinoma, and uncommon histologic variants [177]. Comparing these two most common classification systems, tubular and papillary carcinomas belong to the intestinal type, whereas signet ring carcinoma and other uncommon types correspond to the diffuse type in Lauren’s classification [178]. The intestinal type of GC (IGC) is found in older patients and is mainly caused by *Helicobacter pylori* infection, where carcinoma develops through chronic gastritis and precancerous lesions: atrophic gastritis, intestinal metaplasia, and dysplasia [179]. Additionally, IGC is associated with environmental risk factors such as the consumption of food containing high amounts of salt, cigarette smoking, alcohol intake, sedentary lifestyle and obesity [174]. Moreover, IGC may be found in several hereditary syndromes: Lynch syndrome, Li–Fraumeni syndrome, familial adenomatous polyposis, Peutz–Jaghers syndrome, juvenile polyposis syndrome, hereditary breast and ovarian syndrome, and MUTYH-associated polyposis [180]. The diffuse type of GC (DGC) is observed in younger patients, and it is primarily linked to genetic alterations rather than inflammatory cascade or environmental factors [179]. Hereditary diffuse gastric cancer (hDGC) is the most recognized familial GC, caused by germline mutations in genes: *CDH1* and, rarely, in *CTNNA1* [181]. A few studies showed that miR-1290 plays an important role in GC progression. Lin et al. showed during testing of the synthetic inhibitor of miR-1290 that it targets forkhead box A1 (FOXA1), a member of the FOX transcription factors family, which causes the inhibition of cell proliferation and migration in vitro [182]. Many studies have focused on the role of FOXA1 in human cancers, where its function is tissue specific and it may behave as a tumor suppressor or oncogene [183]. The function of FOXA1 in GC cells is not clear; however, it was shown that FOXA1 induces the expression of E-cadherin and downregulates vimentin, both at the protein level, and thus probably inhibits EMT. Moreover, the upregulation of FOXA1 inhibits GC cells proliferation and tumor formation, and also promotes apoptosis [184]. Further investigation revealed that GC cells incubated with miR-1290 isolated from serum exosomes derived from GC patients showed enhanced proliferation, migration and invasion by targeting naked cuticle homolog 1 (NKD1), an antagonist of the WNT/ß-catenin pathway [185,186]. Other studies demonstrated that GC cells release extracellular vesicles containing miR-1290, which suppress T cell proliferation by targeting transcription factor grainy head-like 2 (Grhl2), resulting in immune escape [187].

#### 7.1.4. Liver Cancer

The most common histologic type of primary liver cancer is hepatocellular carcinoma (HCC), representing 75–85% of cases, and the rest of the liver cancer cases include intrahepatic cholangiocarcinoma and other rare types [188]. Extrinsic risk factors associated with HCC are: hepatotropic viruses (HBV, HCV), alcohol-related cirrhosis, cigarette smoking, obesity, aflatoxins produced by *Aspergillus* species, non-alcoholic fatty liver disease, and liver flukes (*Opisthorchis viverrini* and *Clonorchis sinensis*) [189,190]. Metabolic and genetic diseases that are associated with HCC are: hemochromatosis, Wilson’s disease, alpha-1 antitrypsin disease, glycogen storage disease type I and II, porphyrias, and tyrosinemia [191]. Two major types of genomic instabilities, mitotic error-mediated chromosome instability (CIN) and DNA metabolism defect-mediated microsatellite instability (MIN), are responsible for the heterogeneity of HCC [192]. HCC is associated with genetic alterations in specific chromosomal regions and genes such as mutations of the *TERT* promoter and the deletion of *TP53*, and about 95% of HCC cases showed the deregulation of the Wnt signaling pathway [193]. The better understanding of the molecular basis of this pathology is crucial for the recognition of new molecular targets, treatment decisions and biomarkers [193,194]. HCC is a typical hyper-vascular tumor which uses exosomes enriched in angiogenic factors including miRNAs for cancer progression and metastasis [195]. Recently, it was found that miR-1290 delivered through exosomes from HCC cells to recipient endothelial cells downregulates the suppressor of MEK null (*SMEK1*) expression and promotes tumor angiogenesis [196]. The HCC treatment strategy is a huge challenge, and liver transplantation is one of the therapeutic options to treat the advanced stage of disease, which helps to remove the tumor with the widest margin and surrounding pro-carcinogenic environment [197,198]. It was shown that the level of circulating miR-1290 is significantly upregulated in recipients after HCC recurrence, and also that high levels of miR-1290 are associated with poor OS and disease-free survival (DFS) of patients with HCC after liver transplantation [199]. An effective chemotherapeutic agent, paclitaxel, is applied for treating various types of cancers including HCC through inducing DNA damage and the apoptosis of cancer cells [200]. Yan et al. showed that paclitaxel inhibits the proliferation of HCC cells, stimulates apoptosis and reduces the expression of miR-1290 in vitro [201].

#### 7.1.5. Esophageal Cancer

The main two histologic subtypes of esophageal cancer that originate from the esophageal epithelium are esophageal adenocarcinoma (EA) and ESCC [202,203]. Smoking and drinking alcohol are the main risk factors for ESCC [204]. Other major risk factors are the consumption of hot beverages, the intake of nitrosamines found in pickled vegetables, water and moldy food, achalasia, caustic injury, exposure to ionizing radiation, HPV and HIV infections [202,203,205,206]. Esophageal squamous dysplasia is a validated precursor lesion for ESCC [207]. Familial syndromes associated with ESCC are tylosis, Plummer–Vinson syndrome, and Fanconi anemia [206]. Moreover, increased risk for ESCC is found in heavy drinkers and smokers with deficiency of alcohol dehydrogenase 2 (ALDH2), a key enzyme in the detoxification of acetaldehyde that causes the flushing response to alcohol [208]. Genetic alterations implicated in ESCC are gene mutations (*TP53*, *PIK3CA*, *NOTCH1*, *FAT1*, *FAT2*, *KMT2D*, *ZNF750*), the frequently observed amplification and overexpression of the *CCND1* gene, and the deletion of tumor-suppressor genes such as *TP53, APC*, *CDKN2A*, and *FHIT* [202]. Additionally, a large number of single-nucleotide polymorphisms are associated with ESCC affecting *PLCE1* and *TP53* [209,210]. Epigenetic alterations, including the hypermethylation of promoters of tumor-suppressor genes, genome-wide hypomethylation, histone modifications and miRNAs, also play a significant role in ESCC [211]. It is demonstrated that miR-1290 targets transcription factor nuclear factor I/X (*NFIX*) [130,212]. Comparative analysis between ESCC and matched non-cancerous esophageal mucosa showed higher expression of miR-1290 and markedly lower expression of *NFIX* which is associated with aggressive progression and predict poor prognosis of ESCC patients [130]. In vitro studies confirmed that miR-1290 promotes proliferation, migration and invasion of ESCC cells [212,213]. In addition, high serum levels of miR-1290 may discriminate ESCC patients from normal controls and reflect the progress of ESCC, which suggests a potential diagnostic role of miR-1290 [214].

### 7.2. Lung Cancer

Lung cancer (LC) is the leading cause of cancer-related death, causing about one-quarter of all cancer deaths (over 135,000 estimated deaths in the United States in 2020) [215]. Due to delayed diagnosis, most patients (57%) are diagnosed with metastatic disease and for them the 5-year relative survival rate is very low (5%) [215]. Historically, LC is classified based on tumor histology into NSCLC and small cell lung cancer (SCLC) [216]. NSCLC include several histotypes such as lung adenocarcinoma (LADC), lung squamous cell carcinoma (LSCC), lung adenosquamous carcinoma (LASC), and large cell lung carcinoma (LCLC) [216]. Lung cancer results from multistage carcinogenesis with gradually increasing molecular changes in bronchial epithelium [217]. The first changes are loss of heterozygosity (LOH) or microsatellite instability (MSI), and at the dysplasia stage DNA methylation. The most often mutated proto-oncogenes that drive oncogenic transformation in lung epithelium are oncogenes from the *RAS*, *MYC*, and *HER* families. Among the tumor-suppressor genes that inhibit cellular proliferation and maintain genome stability, the most frequent are the mutations of *TP53*, *CDKN2A*, and *RB*. Epigenetic changes in LC include DNA methylation (hypomethylation or hypermethylation) and disrupted expression of microRNAs (miRNAs) that may inactivate tumor suppressors such as *APC*, *CKDN2A*, *CHD13*, *RARB*, and *RASSF1A* [217]. Bioinformatics analysis of miRNA datasets from the Gene Expression Omnibus (GEO) database revealed hsa-miR-1290 among the top 10 upregulated DE-miRNAs in SCLC compared with normal lung tissue [218]. Similarly, a higher expression of miR-1290 was observed in NSCLC compared with normal adjacent tissues [219]. It was demonstrated that miR-1290 is a crucial driver for tumor initiation and metastasis in NSCLC [220]. MiR-1290 was upregulated in lung cancer stem cells (CSCs) and its loss influenced their tumor-initiating potential and ability to metastasize. Serum miR-1290 levels correlated with the clinical response to therapy of LC patients and the inhibition of miR-1290 with administration of locked nucleic acids arrested the growth of patient-derived xenograft tumors [220]. Furthermore, the suppression of miR-1290 decreased markers of stemness and epithelial–mesenchymal transition (EMT) in NSCLC cell lines [221]. Importantly, anti-miR-1290 inhibited proliferation, colony and sphere-formation, migration, and invasion of NSCLC cells, indicating that miR-1290 plays a role in the invasion and metastasis of NSCLC [221]. Similarly, in vivo experiments showed that antagomir-1290 suppressed NSCLC volume and weight initiated by CD133-positive cells [222]. Furthermore, antagomir-1290 inhibited the proliferation, clonogenicity, migration, and invasion of CD133-positive cells by targeting fyn-related Src family tyrosine kinase [222]. The stimulation of NSCLC cells proliferation, colony formation, and invasion by miR-1290 can be connected with the downregulation of interferon regulatory factor 2 (IRF2), which is a direct target of miR-1290 [223]. Additionally, miR-1290 enhanced the expression of cell-cycle-related proteins such as CDK2 and CDK4, and stimulated EMT by upregulating N-cadherin and downregulating E-cadherin expression [223]. MiR-1290 overexpression enhanced proliferation, cell cycle progression and invasion, while suppressing apoptosis in LADC cells, and miR-1290 was able to stimulate tumor growth, invasion and metastasis in vivo [224]. MiR-1290 activated the PI3K/AKT and JAK/STAT3 pathways by directly targeting the suppressor of cytokine signaling 4 (SOCS4), suggesting that miR-1290 stimulates proliferation invasion, and metastasis of LADC by targeting SOCS4 [224]. The downregulation of miR-1290 by treatment with *Polygonatum odoratum* lectin induced apoptosis and autophagy in A549 LADC cells through the downregulation of the Wnt pathway [225]. Interestingly, miR-1290 sensitized A549 LADC cells to cytotoxicity induced by an anticancer agent, asiatic acid, and decreased the expression of its direct target *BCL2* gene [226]. These findings indicate that miR-1290 is useful as a biomarker and it may become a novel target for miR-1290-based therapies of LC.

The currently used diagnostic strategies, such as chest radiography, sputum cytology, and blood biomarkers (autoantibodies, complement fragments, circulating tumor DNA, carcinoembryonic antigen (CEA), cancer antigen 125 (CA-125), carbohydrate antigen 15.3 (CA15.3), cytokeratin-19 fragment (CYFRA 21-1), neuron-specific enolase (NSE), squamous cell carcinoma antigen (SCCA), and pro-gastrin-releasing peptide (ProGRP)) have insufficient sensitivity or specificity for an early and reliable diagnosis of LC [227]. Thus, novel and effective biomarkers are needed for early diagnosis and screening. Recent studies revealed miR-1290 as a potential biomarker for the diagnosis, chemotherapy response, and clinical outcome of LC patients. miR-1290 expression was increased in NSCLC tissues compared with normal adjacent tissues, and it was positively correlated with tumor stage and lymph node metastasis [131]. Additionally, serum miR-1290 levels were higher in NSCLC patients compared with benign lung disease and healthy individuals. Furthermore, high serum miR-1290 levels predicted shorter survival and miR-1290 was an independent prognostic factor for patients with NSCLC [131]. Similarly, serum exosomal miR-1290 was upregulated in both early- and advanced-stage patients with LADC compared with healthy individuals [132]. In addition, increased levels of serum exosomal miR-1290 were indicated to be tumor-derived because their level decreased after cancer resection. Importantly, exosomal miR-1290 had better diagnostic efficacy than CEA, CYFRA 21-1 and NSE, with an 80.0% sensitivity of and 96.7% specificity. Additionally, LADC patients with higher expression of exosomal miR-1290 had significantly shorter progression-free survival (PFS) and it was demonstrated that serum exosomal miR-1290 is an independent risk factor for LADC prognosis [132]. Interestingly, the latest in vitro study revealed that the irradiation of human bronchial epithelial cells with energetic heavy ions, representative of species found in cosmic rays, stimulated the release of exosomes containing miRNAs involved in cancer initiation and progression, including miR-1290 [228].

Recent studies showed a predictive role of miRNA for chemotherapy response in LC patients [229]. Patients with NSCLC are usually treated with platinum-based chemotherapy, but the response rates are low and no biomarkers that predict response are available. The expression of three miRNAs (miR-1290, miR-196b, and miR-135a*) in LADC tissues predicted a response to the platinum-based doublet chemotherapy in both the test and validation cohort with high accuracy (82.5% and 77.8%, respectively) [230].

### 7.3. Female Cancers

#### 7.3.1. Breast Cancer

Breast cancer (BC) is the most frequent malignant neoplasia in women with over 2.2 million new cases and about 685,000 deaths worldwide in 2020 [1]. BC is a heterogenic group of neoplastic diseases differing in their morphology, molecular pattern, and prognosis, for that reason requiring personalized clinical management [231]. According to molecular characteristics, BC was divided into five intrinsic subtypes. Estrogen receptor (ER)-positive BCs include luminal A tumors—the most common and having the most favorable prognosis—and luminal B tumors—exhibiting higher expression of Ki67 antigen or human epidermal growth factor receptor 2 (HER2) and worse prognosis. Another HER2-overexpressing subtype is more aggressive, ER- and progesterone receptor (PR)-negative, and therefore not sensitive to hormonal therapy, but it can be treated with HER2-targeting drugs such as trastuzumab. Basal-like BCs are predominantly TNBCs that do not express ER, PR, and HER2 and for that reason are not susceptible to both hormonal and anti-HER2 therapy, and usually are characterized by high proliferation rate and poor prognosis. The normal BC, distinguished by a gene expression pattern similar to normal breast epithelium, is a questionable subtype, because it is considered to be a normal cell contamination of a low cancer cell content tumor [231,232]. Further molecular profiling with high-throughput techniques resulted in several extensive and more precise classifications of BC, and one of these alternative approaches is based on a miRNA expression pattern [233]. In spite of comparable characteristics, these new subtypes can differ with clinical outcome; therefore, additional biomarkers for early BC diagnosis, treatment management and prognostic purposes are still required.

Li et al. demonstrated that miR-1290 expression is significantly elevated in exosomes from BC patients compared with healthy groups, but not in patients’ sera [234]. For that reason, the authors concluded that exosomal miRNAs might not be equivalent to serum miRNAs and they have potential to become more valuable biomarkers for BC. This finding was strengthened by the fact that exosomal miR-1290 expression was related to BC progression and it was significantly higher in patients with more advanced stages and in patients with lymph node metastases [234]. Additionally, a previous study by Hamam et al. discovered upregulated plasma or serum miR-1290 in BC patients compared with healthy individuals [235]. Further analysis revealed that its expression was elevated at lower stages, indicating its usefulness in the early detection of BC. MiR-1290 levels increase especially in HER2-positive and TNBC subtypes, which might be explained by the different molecular patterns of BC [235]. The heterogeneity of miR-1290 expression seems to be confirmed by another work where miR-1290 was found to be downregulated in HER2 negative, ER-high, and Ki67-low BCs [236]. Moreover, its expression was significantly correlated with tumor grading and inversely correlated with the expression of its putative targets, N-acetyltransferase-1 (NAT1) and forkhead box A1 (FOXA1), the presence of which is specific for better outcomes for patients with luminal tumors [236]. Indeed, further in vitro study proved that NAT1 is a direct target of miR-1290 in ER-positive BC cells. Moreover, NAT1 was positively correlated with ER and PR expression, negatively correlated with BC tumor grade and size, and associated with longer survival in BC patients treated with tamoxifen and in patients with lymph node metastasis [237]. Interestingly, integrated analysis indicated that miR-1290 was downregulated in TNBC; however, its expression was correlated with poor prognosis, which suggests the high variability of miRNA expression within this subtype [238]. The above conclusion was supported by a significant inverse correlation of miR-1290 with FOXA1, one of the highly upregulated genes in the analyzed TNBC group. These findings suggest that miR-1290 may become a promising prognostic marker for BC.

#### 7.3.2. Cervical Cancer

Cervical cancer (CC) is the world’s most prevalent gynecological cancer, which caused near 342,000 deaths in 2020 [1]. The main risk factor for CC is persistent infection with oncogenic types of human papillomavirus (HPV), leading to almost all of the most common squamous cell carcinomas and the majority of adenocarcinomas [239]. Such high-risk HPVs such as HPV 16 or HPV 18 can drive the premalignant transformation of squamous cells to cervical intraepithelial neoplasia (CIN) that in the long term may evolve into in situ and then invasive carcinomas [240]. Among numerous genetic and epigenetic aberrations which predispose individuals to the development of CC, a deregulated miRNA pattern seems to be important for this process. In addition, the expression of at least several miRNAs could be affected by HPV proteins, or conversely, altered miRNA expression may facilitate the expression of viral proteins [241]. It was also suggested that HPV-induced chronic inflammation, a pivotal process for CC carcinogenesis, may be mediated by miRNAs [242].

Among many deregulated miRNAs in CC, miR-1290 level was significantly elevated in serum from CC patients compared with healthy individuals [133]. Additionally, miR-1290 was gradually increased from the control group, through low- to high-grade CIN, up to the CC group. This association was observed for the CC stage as well, thus demonstrating the usefulness of miR-1290 as an early diagnostic marker [133]. MiR-1290 was also overexpressed in CC cell lines after exposure to radiation, which led to enhanced cell survival, highlighting its role as an oncogenic miR [243]. Because concurrent radiotherapy is a standard treatment of locally advanced CC, miR-1290 has the potential to become a valuable prognostic marker for predicting tumor response to radiotherapy, reinforcing the routinely used evaluation of squamous cell carcinoma antigen [244]. Interestingly, in an in vitro study, Yao et al. found miR-1290 promoter hypermethylation in three CC cell lines with integrated HPV genomes but not in the CC cell line without HPV infection, which may suggest that additional epigenetic regulatory mechanisms are engaged in the modulation of miR-1290 expression in the presence of HPV [245]. However, the methylation status of the miR-1290 promoter was not investigated in CC tissues nor compared with normal cervical cells.

Higher miR-1290 expression was also detected in plasma from endometrial cancer (EC) patients compared with controls, but it was not correlated with clinicopathological characteristics [134]. For that reason, the exact role of miR-1290 in EC needs to be verified by further studies. It was shown that miR-1290 expression differs over the estrous cycle in cows, which may indicate its importance in endometrial remodeling in response to hormonal regulation [92].

#### 7.3.3. Ovarian Cancer

Although the ovarian cancer (OC) worldwide incidence rate in 2020 was third among all gynecological cancers, it has the highest mortality to incidence ratio among all of them [1]. This is mainly caused by the lack of specific symptoms at the early stages of OC and the lack of effective screening methods. For that reason, patients are usually diagnosed at advanced stages and despite the fact that most of them respond well to primary treatment, disease recurrence is frequently observed [246]. Among the few histological types of OC, the worst prognosis and the highest incidence rate are related to high-grade serous cancer (HGSC), which could arise from fallopian tube or ovarian surface epithelium [247]. Other OC histologic subtypes are low-grade serous tumors probably developing from borderline tumors, endometrioid and clear cell carcinomas both originating from endometrial lesions, and mucinous cancers of not well-defined origin. Besides epithelial neoplasms, up to 5% of OCs comprise germ cell and sex-cord tumors, small cell carcinoma and sarcoma [246]. The routine diagnostic serum marker for OC is carbohydrate antigen 125 (CA125), which is useful to evaluate response to treatment and disease relapse; however, its sensitivity at early stages is very limited. Moreover, it may be elevated in the case of other states such as endometriosis, benign cystadenomas, pregnancy, peritoneal inflammation or menstruation [248,249]. The novel and more specific marker is human epididymis protein 4 (HE4), which is overexpressed in OCs, especially in the endometrioid subtype, but it has low expression in the clear cell subtype [249]. Among many currently tested screening strategies for the early detection of OC, one of the main areas of interest is based on circulating miRNAs. It was shown that an appropriate set of several miRNAs has the potential to become a reliable biomarker for the early detection of OC, distinguishing OC from benign, borderline tumors, and other cancers, staging determination, and even subtype classification [250].

A high serum level of miR-1290 appeared to be a useful marker for differentiation between HGSC patients and healthy individuals [135]. Moreover, it allowed HGSC to be distinguished from other histological subtypes of OC and its combination with CA-125 strongly elevated diagnostic accuracy for this most common OC subtype [135]. Many patients with OC suffer from excessive fluid in the peritoneal cavity, which is usually correlated with worse prognosis; therefore, it is reasonable to determine specific markers also in ascites. Indeed, notably increased miR-1290 levels were observed in ascites from patients with different subtypes of OC, especially at advanced stages, which indicates that measurements of miRNAs in ascites may have higher prognostic relevancy than miRNAs evaluated in patients’ blood [136]. Microarray analysis revealed the elevated expression of miR-1290 in tissue samples of recurrent ovarian serous cystadenocarcinoma compared with primary tumor samples [251] and in serous OC compared with control oviduct tissues [252]. On the other hand, there are also studies suggesting that miR-1290 in OC might act as a tumor suppressor. Using an in vitro model, Lai and Cheng found that the direct inhibition of miR-1290 or its sponging by lncRNA CCAT1 promotes the proliferative potential of two OC cell lines, OVCAR-8 and SKOV-3 [253]. Interestingly, another study determined that a high plasma level of miR-1290 in presurgical women with OC predicts favorable overall survival independently from stage and age [254]. Unexpectedly, miR-1290 expression decreased in OC patients after surgical treatment and chemotherapy, but also in long survivors, which may suggest its oncogenic role in OC. However, the above conclusion requires further investigation and the exact role of miR-1290 in OC remains unclear and needs to be validated.

### 7.4. Prostate Cancer

Prostate cancer (PrC) is the second leading cause of cancer-related death in men, with over 190,000 new cases and over 33,000 deaths estimated in the United States in 2020 [215]. The 5-year relative survival rate for all PrC stages combined is high (98%) [215], but many patients with advanced, or metastatic cancer, despite treatment, succumb to the disease [255]. Surgery and radiation therapy are effective for early-stage PrC, but 30–40% of patients will progress to advanced disease that is treated with androgen deprivation [256]. This therapy is initially efficient, but patients will eventually develop incurable castration-resistant prostate cancer (CRPC) [256]. The molecular changes that drive prostate carcinogenesis include the upregulation of anti-apoptotic *BCL-2* and a carcinogen-detoxifying enzyme GSTP1, and the downregulation of tumor suppressors such as *NKX3.1*, *PTEN*, and *CDKN1B* at the proliferative inflammatory atrophy (PIA) stage [257]. The next events include the dysregulation of ETS transcription factor, loss of *NKX3.1*, mutations of *SPOP*, and *TMPRSS2–ERG* fusion at the prostatic intraepithelial neoplasia (PIN) stage. The PrC stage is characterized by the activation of telomerase, overexpression of *MYC*, deletion of tumor suppressors such as *PTEN*, *CDKN1B*, and loss of *RB1* and *TP53* [257]. The accuracy of prostate-specific antigen (PSA) or clinical examination in the screening of PrC is in question, and circulating miRNAs can become alternatives to PrC diagnosis [258]. New biomarkers are needed that will allow the detection of PrC at an early stage and monitor disease progression. The measurements of exosomal miRNA in circulatory fluids offer an attractive potential for predictive and prognostic biomarker development [259]. Initially, slightly reduced miR-1290 expression was detected in PrC tissues compared with benign prostate hyperplasia (BPH) samples, and there was no difference in CRPC compared with BPH [256]. In other study, a higher miR-1290 concentration was found in the serum of men with PrC compared with BPH [260]. Similarly, miR-1290 expression in urinary extracellular vesicles (UEVs) was significantly increased in the PrC patients compared with BPH. However, miR-1290 isolated from UEVs had no prediction value for PrC [260]. Additionally, higher levels of circulating exosomal miR-1290 were associated with shorter overall survival (OS) of patients with castration-resistant prostate cancer (CRPC) [259]. In the latest study, the set of five circulating miRNAs coupled to miR-5100 and miR-1290 reached nearly 99% diagnostic performance for PrC and was superior to a recent 2-miRNA model [258].

### 7.5. Head and Neck Cancers

Head and neck cancers include malignancies of nose, sinuses, oral cavity, pharynx, and larynx.

#### 7.5.1. Oral Cancer

Oral cancer is the most common cancer of the head and neck with over 7000 estimated deaths in the United States in 2020 [215]. Oral squamous cell carcinoma (OSCC) is the major subtype among oral cancers and it is an aggressive neoplasm that spreads to cervical lymph nodes and distant organs [261]. The 5-year survival rates of oral cancer patients are about 50% due to late diagnosis [261]. Oral carcinogenesis is associated with tobacco consumption, alcohol abuse or both and it is a multifactorial process occurring in epithelial cells that are affected by different genetic alterations, including changes in *EGFR*, *TP53*, *NOTCH1*, *CCND1*, *CDKN2A*, *STAT3*, and *RB* [262]. There are no effective preventive measures for OSCC; therefore, early diagnosis is crucial in the treatment of OSCC patients [261]. It was discovered that circulating miR-1290 could be a biomarker for predicting the survival and clinical response to chemoradiotherapy in patients with OSCC. A higher expression of miR-1290 was found in OSCC tissues than that in normal tissues and it was associated with shorter OS [261]. Moreover, the overexpression of miR-1290 enhanced migration, invasion, and EMT in OSCC cells by targeting *CCNG2* [261]. On the other hand, plasma miR-1290 expression was lower in OSCC than that in healthy individuals and low miR-1290 levels were independently associated with shorter OS and PFS [137]. Similarly, plasma levels of miR-1290 were lower in OSCC patients than in the group of healthy volunteers and low miR-1290 levels were present in patients with a poor pathological response for preoperative chemoradiotherapy [138]. Additionally, lower circulating miR-1290 expression was independently associated with shorter OS and DFS [138].

#### 7.5.2. Laryngeal Cancer

Laryngeal cancer (LaC) is a common neoplasm of the head and neck with over 3700 estimated deaths in the United States in 2020 [215]. Despite therapy, the prognosis for LaC is still poor due to lymphatic metastasis, and over the past 40 years the 5-year survival rate of LaC has decreased to 30–40% for advanced disease [263]. Therefore, it is essential to identify novel biomarkers that can predict prognosis and have therapeutic efficacy [264]. The most common subtype of LaC is squamous cell carcinoma (LaSCC), representing around 95% of cases [265]. Central factors participating in LaC development are smoking, alcohol consumption, and HPV infection [265]. The development of LaC is a complex process involving changes in many genes including miRNAs [266]. MiR-1290 was upregulated in supraglottic LaSCC tissues compared with matched normal mucosa [264]. Similarly, miR-1290 was overexpressed in the LaSCC cell lines and in the primary tumors compared with non-malignant controls, and it was able to downregulate the expression of *MAF*, a transcription regulator of epithelial cells differentiation and apoptosis, and *ITPR2*, a regulator of cell apoptosis and senescence [267]. It was found that LaSCC cell line AMC-HN-8 can release exosomes containing several miRNAs including miR-1290 [268] indicating that miR-1290 may become a novel circulating biomarker for LaSCC.

### 7.6. Cutaneous Cancer

Most skin cancers (SC) are nonmelanomatous (SC that originate from keratinized epithelial cells), including the most common squamous cell carcinoma (SCC) [269]. The most common risk factor for SC is exposure to ultraviolet radiation (UVR) and the incidence of SC is increasing as a result of increased exposure to sunlight [270]. UVR causes DNA damage and the development of mutations, inflammation, defective activity of the immune system, and oxidative stress [270]. The genetic landscape of cutaneous SCC (CSCC) includes the alterations of tumor-suppressor genes such as *TP53*, *CDKN2A*, *NOTCH1*, *NOTHC2*, and *p16*; epigenetic regulators including *ARID2*, *CREBBP*, *KMTC2*, *KMT2A*, *SETD2*, and *TET2*; and mutations in DNA repair pathways and in TGF-β receptors. Additionally, several miRNAs are upregulated or downregulated in CSCC, including miRNA-21, -34a, -124, -125b, -148a, -181a, -199a, -205, and -214 [270].

MiR-1290 was found to be upregulated in CSCC tissues compared with healthy skin and its expression was increased in the supernatants of different CSCC cell lines compared with cultured primary keratinocytes [271]. Importantly, miR-1290 serum levels were higher in CSCC patients compared with healthy controls. The upregulation of miR-1290 both in CSCC tissues and in serum suggests that the dysregulation of this miRNA in CSCC tissue might be reflected in the serum of patients with highly differentiated CSCCs and without advanced disease. Therefore, the quantification of miR-1290 in serum may become a tool for the identification of CSCC recurrence and metastasis [271].

### 7.7. Brain Cancer

Brain and other tumors of the nervous system were the leading cause of cancer death in 2017 among young patients (men younger than 40 years and women younger than 20 years) with over 18,020 estimated deaths in 2020 in the United States [215]. Malignant gliomas are aggressive primary brain tumors associated with low quality of life, and increasing relapse and mortality [272]. The OS for patients with high-grade gliomas rarely exceeds 13 months, and the survival of patients with low-grade gliomas is about 5 years. Therefore, it is crucial to discover new biomarkers of the propagation and metastasis of gliomas. The strong invasive potential of malignant gliomas hinders the effect of treatment, and the molecular alterations that drive gliomas’ carcinogenesis process remain unclear [272]. The common molecular changes discovered in gliomas include mutations of *IDH1*, *IDH2*, *TP53*, *RB*, *PTEN*, *PIK3CA*, *PIK3R1*, *NF1*, *ATRX*, and *TERT*-promoter, amplifications of *EGFR*, *PDGFRA*, *CDK4*, *CDK6*, *MDM2*, *MDM4*, and *MET*, gain of chromosome 7, loss of chromosome 10, deletions of 9p21 involving *CDKN2A* and *CDKN2B*, deletion of 19q, *KIAA11549–BRAF* fusion, the upregulation of forkhead box protein M1 gene (*FOXM1*), the activation of E2F2-dependent cell cycle progression, the activation of *MYC* expression, aberrant histone and DNA and methylation, and the epigenetic silencing of genes encoding transcription factors regulated by the polycomb repressive complex 2 [273]. Higher miR-1290 expression was discovered in glioma tissues compared with normal brain samples, and in glioma cell lines compared with normal human glial cells [272]. MiR-1290 promoted the proliferation, migration, and invasion of glioma cells in vitro, and enhanced tumor growth in nude mice. Furthermore, miR-1290 targeted and downregulated the *LHX6* tumor suppressor that inhibits cancer progression through the inhibition of the Wnt/β-catenin pathway [272]. Additionally, miR-1290 increased proliferation, migration, invasion, and resistance to chemo-radiation in the glioblastoma (a high-grade glioma) cells by the suppression of its target *SOCS4*, a member of the SOCS family cytokine-inducible negative regulators [274]. Furthermore, the suppression of miR-1290 increased apoptosis and sensitivity to chemotherapy drugs, suggesting that miR-1290 may become a new target for the clinical therapy of glioblastoma [274]. MiR-1290 was upregulated in glioma tissues compared with normal brain samples and it suppressed the *FBXW7* tumor suppressor [275]. Another study showed that miR-1290 promotes a malignant phenotype of glioma cells by targeting the *RORA* tumor suppressor [276]. Interestingly, miR-1290 was upregulated in exosomes released by glioma stem cells (GSCs) compared with exosomes from normal neural stem cells (NSCs), and exosomes secreted by GSCs influenced the gene expression in receiving NSCs, including genes with a role in tumorigenesis [277]. The findings of these studies provide new insights into the possible functions of the exosomes in the regulation of normal cell behavior, and suggest the diagnostic or prognostic value of miR-1290 in gliomas.

### 7.8. Leukemia

Leukemia is the second leading cause of cancer death among young patients (men younger than 40 years and women younger than 20 years) with over 23,000 estimated deaths in 2020 in the United States [215]. Acute lymphoblastic leukemia (ALL) is a common childhood hematologic malignancy with a 5-year event-free survival (EFS) of 63–83% in pediatric cases, and 40% in adult patients [278]. Numerous patients with ALL suffer from the adverse effects caused by conventional chemotherapy and die from relapsed disease [278]. Large-scale genomic studies identified recurrent genomic changes enriched in relapsed ALL such as *CREBBP*, *IKZF1*, *NT5C2*, *PRPS1*, *SETD2*, *TP53* [279]. Some of these genetic alterations are associated with resistance to therapy, e.g., mutations in *CREBBP* and *IKZF1* with glucocorticoid resistance, and mutations in *PRPS1* and *NT5C2* with thiopurine treatment [279]. Therefore, novel treatment strategies are needed to improve the clinical outcome of patients with ALL. Resveratrol, a natural polyphenol, inhibited proliferation and migration, and induced cell cycle arrest and apoptosis in a B-cell ALL (B-ALL) cell line by decreasing the overexpression of miR-1290 [278]. Moreover, high miR-1290 expression in bone marrow aspirates predicted shorter relapse-free survival (RFS) in pediatric patients with B-ALL [280].

### 7.9. Conjunctival Melanoma

Conjunctival melanoma (CM) is a rare ocular malignancy with an approximately 30% 10-year mortality rate [281]. Genetic studies have identified mutations in the *BRAF*, *NF1*, *NRAS*, *KIT*, and in the *TERT* genes promoters suggesting a close relation of CM with skin melanoma rather than with uveal melanoma. There is an urgent need to discover novel biomarkers that may predict the clinical course of the CM. Interestingly, hsa-miR-1290 was downregulated in tissues of primary CM with metastatic spread compared with nonmetastatic tumors [281].

## 8. Conclusions and Future Perspectives

The ever-increasing number of published findings unquestionably points to the role of miR-1290 in the pathogenesis of different types of cancer. The deregulated expression of miR-1290 promotes tumor initiation, growth, survival, angiogenesis and metastasis. In addition to the current knowledge of tumorigenesis, it was also confirmed that miR-1290 is a useful biomarker for cancer diagnosis and tumor staging, and it may also serve as a predictor of cancer treatment, disease recurrence, and patient outcome. For the above reasons, using miR-1290 as a marker in routine clinical practice is a huge challenge in the future. Despite numerous promising reports, fundamental issues should be resolved before the future implementation of this powerful marker in medical diagnostics. The major limitation in the utility of miR-1290 is the selection of the most appropriate type of sample obtained from the patient. It is still not clarified whether the best choice for assessing and monitoring the expression of miR-1290 is tissue samples, blood samples or exosomes isolated from the blood. Last but not least, another issue is the diverse function of miR-1290 in different types of cancers. Although in the vast majority of studies, it exerts an oncogenic function, several reports confirm its tumor-suppressive role. In order to overcome these problems, further investigations are needed to clarify the biological role of miR-1290 in different neoplasms and to introduce it as a biomarker used in clinical practice in the near future.

## Figures and Tables

**Table 1:** Clinical value of miR-1290 in neoplastic disease.

Type of Cancer	Source of miR-1290	Adjuvant Therapy	Clinical Significance of miR-1290	References
Colorectal cancer	Stage II and III CRC tissues (n = 291) resected before adjuvant therapy	5-FU-based chemotherapy after radical resection	miR-1290 is an independent prognostic factor for OS (HR = 1.48; 95% CI: 0.85–2.90) and DFS (HR = 1.59; 95% CI: 1.32–2.95).	[123]
	Plasma from stage I-IV CRC patients (n = 80) collected before adjuvant therapy, colorectal adenoma (n = 50), and HCs (n = 30)	Not indicated	miR-1290 can differentiate colorectal adenoma from HCs with an AUC = 0.78 (95% CI: 0.69–0.88), 75.53% SE and 87.41% SP, and CRC patients from HCs with an AUC = 0.88 (95% CI: 0.82–0.95), 76.65% SE and 90.23% SP.	[124]
	Serum exosomes from stage I-IV CRC patients (n = 100) collected before adjuvant therapy and HCs (n = 35)	Not indicated	miR-1290 can discriminate stage I CRC patients from HCs with an AUC = 0.89 (95% CI: 0.81–0.97), 83.33% SE and 85.71% SP, and CRC patients at different stages from HCs with an AUC = 0.92 (95% CI: 0.87–0.97), 85% SE and 88.57% SP.	[125]
	Stage I-IV CRC tissues (n = 179) resected before adjuvant therapy. Preoperative sera from stage I-IV CRC patients (n = 211), colorectal adenoma (n = 56), and HCs (n = 57)	5-FU-based chemotherapy for patients with stage III/IV CRC, no adjuvant therapy to stage I/II CRC patients	Tissue miR-1290 is not an independent prognostic factor for OS. Serum miR-1290 can distinguish colorectal adenoma from HCs with an AUC = 0.72, 46.4% SE and 91.2% SP, and CRC patients from HCs with an AUC = 0.83, 70.1% SE and 91.2% SP. Serum miR-1290 is an independent prognostic marker for OS (HR = 4.51; 95% CI: 1.23–23.69) and an independent predictor for tumor recurrence after curative surgery (HR = 3.92; 95% CI: 1.11–25.14).	[126]
Pancreatic cancer	Preoperative sera from patients with stage I-III PanC (n = 41), stage I-III pancreatic neuroendocrine tumors (n = 18), CP (n = 35), and HCs (n = 19). Preoperative sera from patients with PanC (n = 56).	Pancreatic resection followed by 5-FU-based and palliative therapies (including gemcitabine)	miR-1290 can discriminate subjects with PanC relative to HCs, CP, and pancreatic neuroendocrine tumors with an AUC = 0.96 (95% CI: 0.91–1.00), 0.81 (0.71–0.91), and 0.80 (0.67–0.93), respectively. miR-1290 is an independent prognostic biomarker for OS (HR = 2.24; 95% CI: 1.16–4.33).	[127]
	Plasma from stage I-IV PanC patients (n = 167, collected before surgery or before chemotherapy for patients with advanced disease) and HCs (n = 267)	Adjuvant chemotherapy administered to 61% of patients	miR-1290 can discriminate patients with PanC from HCs with an AUC = 0.73 (95% CI: 0.68–0.79), 56.3% SE and 89.5% SP. miR-1290 is not an independent prognostic factor for OS and DFS.	[128]
	Sera from stage I-IV PanC patients (n = 120, obtained before any therapeutic procedures), benign pancreatic disease controls (n = 40), and HCs (n = 40)	Not indicated	miR-1290 can discriminate PanC from HCs and benign controls with an AUC = 0.93 (95% CI: 0.89–0.97), 75.0% SE and 97.5% SP, and an AUC = 0.89 (95% CI: 0.84–0.94), 88.3% SE and 72.5% SP, respectively. miR-1290 is an independent risk factor for PanC (OR = 12.35).	[129]
Esophageal cancer	ESCC tissues (n = 100) resected before adjuvant therapy	Not indicated	miR-1290 is an independent prognostic factor for OS (HR = 1.97; 95% CI: 1.00–4.19) and DFS (HR = 1.81; 1.00–4.06).	[130]
Lung cancer	Sera of stage I-IV NSCLC patients (n = 66) collected before any therapeutic procedures	Not indicated	miR-1290 is an independent prognostic factor for OS (HR = 1.79; 95% CI: 1.17–2.98).	[131]
	Serum exosomes from stage I-IV LADC patients (n = 60) collected before any antitumor therapy	Not indicated	miR-1290 can discriminate LADC from HCs with an AUC = 0.94 (95% CI: 0.89–0.99), 80.0% SE and 96.7% SP. miR-1290 is an independent predictor of PFS (HR = 7.80, 95% CI: 1.44–42.41).	[132]
Cervical cancer	Sera of stage I-IV CC patients (n = 45, collected before adjuvant therapy), 55 CIN, and 31 HCs	Not indicated	miR-1290 can differentiate subjects with CC from HCs with an AUC of 0.80 (95% CI: 0.69–0.90), 90.3% SE and 62.2% SP.	[133]
Endometrioid endometrial carcinoma	Plasma of stage I-IV EEC patients (n = 34) and HCs (n = 14)	Radiotherapy and/or chemotherapy	miR-1290 can discriminate subjects with EEC from HCs with an AUC = 0.77 (95% CI: 0.63–0.88), 76% SE and 86% SP.	[134]
Ovarian cancer	Sera from stage I-IV OC patients (n = 70; including HGSOC, n = 30) and HCs (n = 13)	Not indicated	miR-1290 can distinguish OC patients from HCs with an AUC = 0.48, 51% SE and 57% SP, and HGSOC patients from HCs with an AUC = 0.71, 63% SE and 85% SP.	[135]
	Ascitic fluid or peritoneal lavages from stage I-IV OC patients (n = 23) and plasma from HCs (n = 34)	Not indicated	miR-1290 can discriminate OC patients from HCs with an AUC = 1.00.	[136]
Oral cancer	Plasma from OSCC patients (n = 70) collected before adjuvant therapy and HCs (n = 40)	Not indicated	miR-1290 can distinguish OSCC patients from HCs with an AUC = 0.90 (95% CI: 0.84–0.96), 89.2% SE and 85.0% SP. Low miR-1290 level is an independent risk factor for the poor prognosis (HR = 2.74, 95% CI: 2.15–6.12).	[137]
	Preoperative plasma from stage II-IV OSCC patients (n = 55)	Preoperative 5-FU-based chemoradiotherapy	Low miR-1290 level is an independent predictor of OS (HR = 3.35, 95% CI: 1.08–12.0) and DFS (HR = 3.31, 95% CI: 1.08–11.6).	[138]

Legend: CRC—colorectal cancer; 5-FU—5-fluorouracil; OS—overall survival; DFS—disease-free survival; HR—hazard ratio of multivariate Cox proportional hazard regression analysis; CI—confidence interval; AUC—area under the curve of ROC analysis; SE—sensitivity; SP—specificity; HCs—healthy controls; PanC—pancreatic cancer; CP—chronic pancreatitis; ESCC—esophageal squamous cell carcinoma; NSCLC—non-small cell lung cancer; LADC—lung adenocarcinoma; PFS—progression-free survival; CC—cervical cancer; CIN—cervical intraepithelial neoplasia; EEC—endometrioid endometrial carcinoma; OC—ovarian cancer; HGSOC—high-grade serous ovarian cancer; OSCC—oral squamous cell carcinoma.

## Data Availability

No new data were created and analyzed in this manuscript. Data sharing is not applicable.

## Supplementary Materials

The following are available online at https://www.mdpi.com/article/10.3390/ijms23031234/s1.
