# Supplementary material for: An Insight into miR-1290: An Oncogenic miRNA with Diagnostic Potential
Source: Int J Mol Sci. 2022 Jan 22;23(3):1234. doi: 10.3390/ijms23031234 (PMC8835968; doi:10.3390/ijms23031234)
Supplement: Supplementary file 1 [file ijms-23-01234-s001.zip › ijms-1511389-supplementary.pdf]

Supplementary Table S1. MiR-1290 target genes predicted with miRWalk 3.0 and miRDB.

Gene symbol	Entrez ID	Description
A1CF	29974	Homo sapiens APOBEC1 complementation factor (A1CF), transcript variant 4, mRNA.
ABCC4	10257	Homo sapiens ATP binding cassette subfamily C member 4 (ABCC4), transcript variant 2, mRNA.
ABLIM1	3983	Homo sapiens actin binding LIM protein 1 (ABLIM1), transcript variant 2, mRNA.
ACSL4	2182	Homo sapiens acyl-CoA synthetase long chain family member 4(ACSL4), transcript variant 3, mRNA.
ADARB2	105	Homo sapiens adenosine deaminase RNA specific B2 (inactive)(ADARB2), mRNA.
AGO1	26523	Homo sapiens argonaute RISC component 1 (AGO1), transcript variant1, mRNA.
AKR7A2	8574	Homo sapiens aldo-keto reductase family 7 member A2 (AKR7A2), transcript variant 2, mRNA.
ANK2	287	Homo sapiens ankyrin 2 (ANK2), transcript variant 3, mRNA.
ANKRD12	23253	Homo sapiens ankyrin repeat domain 12 (ANKRD12), transcript variant2, mRNA.
ANKRD42	338699	Homo sapiens ankyrin repeat domain 42 (ANKRD42), transcript variant3, mRNA.
ANTXR2	118429	Homo sapiens ANTXR cell adhesion molecule 2 (ANTXR2), transcript variant 2, mRNA.
AP3B1	8546	Homo sapiens adaptor related protein complex 3 subunit beta 1(AP3B1), transcript variant 2, mRNA.
ARHGAP17	55114	Homo sapiens Rho GTPase activating protein 17 (ARHGAP17), transcript variant 1, mRNA.
ARHGAP5	394	Homo sapiens Rho GTPase activating protein 5 (ARHGAP5), transcript variant 1, mRNA.
ARID4B	51742	Homo sapiens AT-rich interaction domain 4B (ARID4B), transcript variant 3, mRNA.
ASXL3	80816	Homo sapiens ASXL transcriptional regulator 3 (ASXL3), mRNA.
ATF1	466	Homo sapiens activating transcription factor 1 (ATF1), mRNA.
ATF7IP	55729	Homo sapiens activating transcription factor 7 interacting protein (ATF7IP), transcript variant 3, mRNA.
ATP2B1	490	Homo sapiens ATPase plasma membrane Ca2+ transporting 1 (ATP2B1), transcript variant 1, mRNA.
ATP7B	540	Homo sapiens ATPase copper transporting beta (ATP7B), transcript variant 1, mRNA.
ATXN1	6310	Homo sapiens ataxin 1 (ATXN1), transcript variant 1, mRNA.
BBS9	27241	Homo sapiens Bardet-Biedl syndrome 9 (BBS9), transcript variant 3, mRNA.
BBX	56987	Homo sapiens BBX high mobility group box domain containing (BBX), transcript variant 1, mRNA.
BEND4	389206	Homo sapiens BEN domain containing 4 (BEND4), transcript variant 2, mRNA.
BICD2	23299	Homo sapiens BICD cargo adaptor 2 (BICD2), transcript variant 1, mRNA.
BMP3	651	Homo sapiens bone morphogenetic protein 3 (BMP3), mRNA.
BNC2	54796	Homo sapiens basonuclin 2 (BNC2), transcript variant 2, mRNA.
BRWD1	54014	Homo sapiens bromodomain and WD repeat domain containing 1 (BRWD1), transcript variant 3, mRNA.
BTD	686	Homo sapiens biotinidase (BTD), transcript variant 1, mRNA.
C1orf52	148423	Homo sapiens chromosome 1 open reading frame 52 (C1orf52), transcript variant 1, mRNA.
CACNB4	785	Homo sapiens calcium voltage-gated channel auxiliary subunit beta 4(CACNB4), transcript variant 2, mRNA.
CALCR	799	Homo sapiens calcitonin receptor (CALCR), transcript variant 1, mRNA.
CAMK2A	815	Homo sapiens calcium/calmodulin dependent protein kinase II alpha (CAMK2A), transcript variant 3, mRNA.
CAMK4	814	Homo sapiens calcium/calmodulin dependent protein kinase IV(CAMK4), transcript variant 2, mRNA.
CAPN2	824	Homo sapiens calpain 2 (CAPN2), transcript variant 2, mRNA.
CBFA2T3	863	Homo sapiens CBFA2/RUNX1 partner transcriptional co-repressor 3(CBFA2T3), transcript variant 1, mRNA.
CCDC141	285025	Homo sapiens coiled-coil domain containing 141 (CCDC141), transcript variant 2, mRNA.
CCDC142	84865	Homo sapiens coiled-coil domain containing 142 (CCDC142), transcript variant 1, mRNA.
CCDC186	55088	Homo sapiens coiled-coil domain containing 186 (CCDC186), transcript variant 3, mRNA.
CCPG1	9236	Homo sapiens cell cycle progression 1 (CCPG1), transcript variant3, mRNA.
CD164	8763	Homo sapiens CD164 molecule (CD164), transcript variant 2, mRNA.
CD1E	913	Homo sapiens CD1e molecule (CD1E), transcript variant 2, mRNA.
CD4	920	Homo sapiens CD4 molecule (CD4), transcript variant 1, mRNA.
CDC5L	988	Homo sapiens cell division cycle 5 like (CDC5L), mRNA.
CDYL2	124359	Homo sapiens chromodomain Y like 2 (CDYL2), mRNA.
CHN2	1124	Homo sapiens chimerin 2 (CHN2), transcript variant 1, mRNA.
CHST11	50515	Homo sapiens carbohydrate sulfotransferase 11 (CHST11), transcript variant 2, mRNA.
CHTF8	54921	Homo sapiens chromosome transmission fidelity factor 8 (CHTF8), transcript variant 1, mRNA.
CLIP3	25999	Homo sapiens CAP-Gly domain containing linker protein 3 (CLIP3), transcript variant 1, mRNA.
CNOT7	29883	Homo sapiens CCR4-NOT transcription complex subunit 7 (CNOT7), transcript variant 3, mRNA.
CNTN6	27255	Homo sapiens contactin 6 (CNTN6), transcript variant 2, mRNA.
COA8	84334	Homo sapiens cytochrome c oxidase assembly factor 8 (COA8), transcript variant 2, mRNA.
COL21A1	81578	Homo sapiens collagen type XXI alpha 1 chain (COL21A1), transcript variant 2, mRNA.
COLEC12	81035	Homo sapiens collectin subfamily member 12 (COLEC12), mRNA.
CPEB2	132864	Homo sapiens cytoplasmic polyadenylation element binding protein 2(CPEB2), transcript variant C, mRNA.
CREB5	9586	Homo sapiens cAMP responsive element binding protein 5 (CREB5), transcript variant 4, mRNA.
CSNK1G1	53944	Homo sapiens casein kinase 1 gamma 1 (CSNK1G1), transcript variant2, mRNA.
CTDP1	9150	Homo sapiens CTD phosphatase subunit 1 (CTDP1), transcript variant3, mRNA.
CTDSPL	10217	Homo sapiens CTD small phosphatase like (CTDSPL), transcript variant 1, mRNA.
CTTNBP2NL	55917	Homo sapiens CTTNBP2 N-terminal like (CTTNBP2NL), mRNA.
CUL2	8453	Homo sapiens cullin 2 (CUL2), transcript variant 4, mRNA.
CUL4A	8451	Homo sapiens cullin 4A (CUL4A), transcript variant 1, mRNA.
CXorf66	347487	Homo sapiens chromosome X open reading frame 66 (CXorf66), mRNA.

CYP4F11	57834	Homo sapiens cytochrome P450 family 4 subfamily F member 11(CYP4F11), transcript variant 2, mRNA.
DCC	1630	Homo sapiens DCC netrin 1 receptor (DCC), mRNA.
DCX	1641	Homo sapiens doublecortin (DCX), transcript variant 1, mRNA.
DHX33	56919	Homo sapiens DEAH-box helicase 33 (DHX33), transcript variant 2, mRNA.
DLG1	1739	Homo sapiens discs large MAGUK scaffold protein 1 (DLG1), transcript variant 1, mRNA.
DNAJC12	56521	Homo sapiens DnaJ heat shock protein family (Hsp40) member C12(DNAJC12), transcript variant 1, mRNA.
DTL	51514	Homo sapiens denticleless E3 ubiquitin protein ligase homolog (DTL), transcript variant 2, mRNA.
DYNC1I1	1780	Homo sapiens dynein cytoplasmic 1 intermediate chain 1 (DYNC1I1), transcript variant 2, mRNA.
DYRK1A	1859	Homo sapiens dual specificity tyrosine phosphorylation regulated kinase 1A (DYRK1A), transcript variant 6, mRNA.
EBF1	1879	Homo sapiens EBF transcription factor 1 (EBF1), transcript variant1, mRNA.
EGFR	1956	Homo sapiens epidermal growth factor receptor (EGFR), transcript variant 5, mRNA.
ELF5	2001	Homo sapiens E74 like ETS transcription factor 5 (ELF5), transcript variant 3, mRNA.
ELMOD2	255520	Homo sapiens ELMO domain containing 2 (ELMOD2), mRNA.
EME1	146956	Homo sapiens essential meiotic structure-specific endonuclease 1(EME1), transcript variant 1, mRNA.
ENSA	2029	Homo sapiens endosulfine alpha (ENSA), transcript variant 3, mRNA.
ERBB4	2066	Homo sapiens erb-b2 receptor tyrosine kinase 4 (ERBB4), transcript variant JM-a/CVT-2, mRNA.
ERCC4	2072	Homo sapiens ERCC excision repair 4, endonuclease catalytic subunit (ERCC4), mRNA.
ERCC6L2	375748	Homo sapiens ERCC excision repair 6 like 2 (ERCC6L2), transcript variant 3, mRNA.
ESRRB	2103	Homo sapiens estrogen related receptor beta (ESRRB), transcript variant 2, mRNA.
ETV3	2117	Homo sapiens ETS variant transcription factor 3 (ETV3), transcript variant 1, mRNA.
FAM227A	646851	Homo sapiens family with sequence similarity 227 member A(FAM227A), transcript variant 1, mRNA.
FAM53C	51307	Homo sapiens family with sequence similarity 53 member C (FAM53C), transcript variant 1, mRNA.
FAT3	120114	Homo sapiens FAT atypical cadherin 3 (FAT3), transcript variant 2, mRNA.
FAXC	84553	Homo sapiens failed axon connections homolog, metaxin like GST domain containing (FAXC), transcript variant 2, mRNA.
FBXO27	126433	Homo sapiens F-box protein 27 (FBXO27), mRNA.
FCHO2	115548	Homo sapiens FCH and mu domain containing endocytic adaptor 2(FCHO2), transcript variant 2, mRNA.
FCRL5	83416	Homo sapiens Fc receptor like 5 (FCRL5), transcript variant 2, mRNA.
FGF12	2257	Homo sapiens fibroblast growth factor 12 (FGF12), transcript variant 3, mRNA.
FGF5	2250	Homo sapiens fibroblast growth factor 5 (FGF5), transcript variant3, mRNA.
FHL5	9457	Homo sapiens four and a half LIM domains 5 (FHL5), transcript variant 2, mRNA.
FMN1	342184	Homo sapiens formin 1 (FMN1), transcript variant 2, mRNA.
FOXC1	2296	Homo sapiens forkhead box C1 (FOXC1), mRNA.
FREM1	158326	Homo sapiens FRAS1 related extracellular matrix 1 (FREM1), transcript variant 2, mRNA.
FRMD7	90167	Homo sapiens FERM domain containing 7 (FRMD7), transcript variant2, mRNA.
FUT9	10690	Homo sapiens fucosyltransferase 9 (FUT9), mRNA.
FYB1	2533	Homo sapiens FYN binding protein 1 (FYB1), transcript variant 3, mRNA.
GALNT10	55568	Homo sapiens polypeptide N-acetylgalactosaminyltransferase 10(GALNT10), mRNA.
GALNT15	117248	Homo sapiens polypeptide N-acetylgalactosaminyltransferase 15(GALNT15), transcript variant 2, mRNA.
GANC	2595	Homo sapiens glucosidase alpha, neutral C (GANC), transcript variant 2, mRNA.
GAS2L3	283431	Homo sapiens growth arrest specific 2 like 3 (GAS2L3), transcript variant 2, mRNA.
GFRA1	2674	Homo sapiens GDNF family receptor alpha 1 (GFRA1), transcript variant 3, mRNA.
GLIPR1	11010	Homo sapiens GLI pathogenesis related 1 (GLIPR1), mRNA.
GLIS3	169792	Homo sapiens GLIS family zinc finger 3 (GLIS3), transcript variant1, mRNA.
GPR161	23432	Homo sapiens G protein-coupled receptor 161 (GPR161), transcript variant 1, mRNA.
GRAMD2A	196996	Homo sapiens GRAM domain containing 2A (GRAMD2A), mRNA.
GRHL1	29841	Homo sapiens grainyhead like transcription factor 1 (GRHL1), mRNA.
GRM5	2915	Homo sapiens glutamate metabotropic receptor 5 (GRM5), transcript variant b, mRNA.
GSTCD	79807	Homo sapiens glutathione S-transferase C-terminal domain containing (GSTCD), transcript variant 1, mRNA.
GTF2H3	2967	Homo sapiens general transcription factor IIH subunit 3 (GTF2H3), transcript variant 2, mRNA.
HGF	3082	Homo sapiens hepatocyte growth factor (HGF), transcript variant 1, mRNA.
HIGD2A	192286	Homo sapiens HIG1 hypoxia inducible domain family member 2A(HIGD2A), mRNA; nuclear gene for mitochondrial product.
HNRNPF	3185	Homo sapiens heterogeneous nuclear ribonucleoprotein F (HNRNPF), transcript variant 2, mRNA.
HTRA3	94031	Homo sapiens HtrA serine peptidase 3 (HTRA3), transcript variant 2, mRNA.
HYDIN	54768	Homo sapiens HYDIN axonemal central pair apparatus protein (HYDIN), transcript variant 3, mRNA.
IFIT1	3434	Homo sapiens interferon induced protein with tetratricopeptide repeats 1 (IFIT1), transcript variant 2, mRNA.
IL5RA	3568	Homo sapiens interleukin 5 receptor subunit alpha (IL5RA), transcript variant 1, mRNA.
IRAK3	11213	Homo sapiens interleukin 1 receptor associated kinase 3 (IRAK3), transcript variant 2, mRNA.
KBTBD11	9920	Homo sapiens kelch repeat and BTB domain containing 11 (KBTBD11), mRNA.
KCNK2	3776	Homo sapiens potassium two pore domain channel subfamily K member 2(KCNK2), transcript variant 1, mRNA.
KCNMA1	3778	Homo sapiens potassium calcium-activated channel subfamily M alpha1 (KCNMA1), transcript variant 1, mRNA.
KCTD16	57528	Homo sapiens potassium channel tetramerization domain containing 16(KCTD16), transcript variant 1, mRNA.
KDM5A	5927	Homo sapiens lysine demethylase 5A (KDM5A), mRNA.
KIAA1210	57481	Homo sapiens KIAA1210 (KIAA1210), transcript variant 1, mRNA.
KIAA1522	57648	Homo sapiens KIAA1522 (KIAA1522), transcript variant 2, mRNA.

KIF13B	23303	Homo sapiens kinesin family member 13B (KIF13B), mRNA.
KLC1	3831	Homo sapiens kinesin light chain 1 (KLC1), transcript variant 3, mRNA.
KLHL6	89857	Homo sapiens kelch like family member 6 (KLHL6), mRNA.
KLK7	5650	Homo sapiens kallikrein related peptidase 7 (KLK7), transcript variant 3, mRNA.
KMT2D	8085	Homo sapiens lysine methyltransferase 2D (KMT2D), mRNA.
KPNA6	23633	Homo sapiens karyopherin subunit alpha 6 (KPNA6), mRNA.
KPNB1	3837	Homo sapiens karyopherin subunit beta 1 (KPNB1), transcript variant2, mRNA.
L2HGDH	79944	Homo sapiens L-2-hydroxyglutarate dehydrogenase (L2HGDH), mRNA;nuclear gene for mitochondrial product.
LAMA3	3909	Homo sapiens laminin subunit alpha 3 (LAMA3), transcript variant 2, mRNA.
LDHA	3939	Homo sapiens lactate dehydrogenase A (LDHA), transcript variant 2, mRNA.
LNPK	80856	Homo sapiens lunapark, ER junction formation factor (LNPK), transcript variant 1, mRNA.
LPP	4026	Homo sapiens LIM domain containing preferred translocation partnerin lipoma (LPP), transcript variant 2, mRNA.
LRRC8C	84230	Homo sapiens leucine rich repeat containing 8 VRAC subunit C(LRRC8C), mRNA.
LRRTM3	347731	Homo sapiens leucine rich repeat transmembrane neuronal 3 (LRRTM3), transcript variant 1, mRNA.
MAGOH	4116	Homo sapiens mago homolog, exon junction complex subunit (MAGOH), mRNA.
MAP3K2	10746	Homo sapiens mitogen-activated protein kinase kinase kinase 2(MAP3K2), transcript variant 2, mRNA.
MAP3K20	51776	Homo sapiens mitogen-activated protein kinase kinase kinase 20(MAP3K20), transcript variant 1, mRNA.
MAP3K7	6885	Homo sapiens mitogen-activated protein kinase kinase kinase 7(MAP3K7), transcript variant A, mRNA.
MAPK6	5597	Homo sapiens mitogen-activated protein kinase 6 (MAPK6), mRNA.
MARCHF6	10299	Homo sapiens membrane associated ring-CH-type finger 6 (MARCHF6), transcript variant 2, mRNA.
MBNL2	10150	Homo sapiens muscleblind like splicing regulator 2 (MBNL2), transcript variant 4, mRNA.
MDM1	56890	Homo sapiens Mdm1 nuclear protein (MDM1), transcript variant 4, mRNA.
MDM4	4194	Homo sapiens MDM4 regulator of p53 (MDM4), transcript variant 2, mRNA.
MMD2	221938	Homo sapiens monocyte to macrophage differentiation associated 2(MMD2), transcript variant 1, mRNA.
MMP16	4325	Homo sapiens matrix metallopeptidase 16 (MMP16), mRNA.
MOB1A	55233	Homo sapiens MOB kinase activator 1A (MOB1A), transcript variant 2, mRNA.
MS4A10	341116	Homo sapiens membrane spanning 4-domains A10 (MS4A10), mRNA.
MS4A3	932	Homo sapiens membrane spanning 4-domains A3 (MS4A3), transcript variant 3, mRNA.
MSI2	124540	Homo sapiens musashi RNA binding protein 2 (MSI2), transcript variant 3, mRNA.
MSR1	4481	Homo sapiens macrophage scavenger receptor 1 (MSR1), transcript variant 4, mRNA.
MSTN	2660	Homo sapiens myostatin (MSTN), mRNA.
MTHFSD	64779	Homo sapiens methenyltetrahydrofolate synthetase domain containing (MTHFSD), transcript variant 1, mRNA.
MUC21	394263	Homo sapiens mucin 21, cell surface associated (MUC21), transcript variant 1, mRNA.
NAPEPLD	222236	Homo sapiens N-acyl phosphatidylethanolamine phospholipase D(NAPEPLD), transcript variant 1, mRNA.
NAV1	89796	Homo sapiens neuron navigator 1 (NAV1), transcript variant 2, mRNA.
NCOA2	10499	Homo sapiens nuclear receptor coactivator 2 (NCOA2), transcript variant 1, mRNA.
NDUFA5	4698	Homo sapiens NADH:ubiquinone oxidoreductase subunit A5 (NDUFA5),transcript variant 2, mRNA.
NEK11	79858	Homo sapiens NIMA related kinase 11 (NEK11), transcript variant 3, mRNA.
NIPSNAP2	2631	Homo sapiens nipsnap homolog 2 (NIPSNAP2), transcript variant 2, mRNA.
NIPSNAP3B	55335	Homo sapiens nipsnap homolog 3B (NIPSNAP3B), transcript variant 1, mRNA.
NKD1	85407	Homo sapiens NKD inhibitor of WNT signaling pathway 1 (NKD1), mRNA.
NLGN3	54413	Homo sapiens neuroligin 3 (NLGN3), transcript variant 3, mRNA.
NLK	51701	Homo sapiens nemo like kinase (NLK), mRNA.
NLRC3	197358	Homo sapiens NLR family CARD domain containing 3 (NLRC3), transcript variant 1, mRNA.
NOTCH1	4851	Homo sapiens notch receptor 1 (NOTCH1), mRNA.
NRAS	4893	Homo sapiens NRAS proto-oncogene, GTPase (NRAS), mRNA.
NRP2	8828	Homo sapiens neuropilin 2 (NRP2), transcript variant 2, mRNA.
NRXN1	9378	Homo sapiens neurexin 1 (NRXN1), transcript variant alpha2, mRNA.
NUAK1	9891	Homo sapiens NUAK family kinase 1 (NUAK1), mRNA.
NXPE3	91775	Homo sapiens neurexophilin and PC-esterase domain family member 3(NXPE3), transcript variant 1, mRNA.
ODR4	54953	Homo sapiens odr-4 GPCR localization factor homolog (ODR4), transcript variant 2, mRNA.
OPRM1	4988	Homo sapiens opioid receptor mu 1 (OPRM1), transcript variantMOR-1, mRNA.
OSBPL6	114880	Homo sapiens oxysterol binding protein like 6 (OSBPL6), transcript variant 3, mRNA.
OTUD4	54726	Homo sapiens OTU deubiquitinase 4 (OTUD4), transcript variant 3, mRNA.
PALM2AKAP2	445815	Homo sapiens PALM2 and AKAP2 fusion (PALM2AKAP2), transcript variant 5, mRNA.
PANK1	53354	Homo sapiens pantothenate kinase 1 (PANK1), transcript variant gamma, mRNA.
PARP8	79668	Homo sapiens poly(ADP-ribose) polymerase family member 8 (PARP8),transcript variant 1, mRNA.
PARP8	79668	Homo sapiens poly(ADP-ribose) polymerase family member 8 (PARP8),transcript variant 1, mRNA.
PARVA	55742	Homo sapiens parvin alpha (PARVA), mRNA.
PCMTD2	55251	Homo sapiens protein-L-isoaspartate (D-aspartate) O-methyltransferase domain containing 2 (PCMTD2), transcript
PDS5A	23244	Homo sapiens PDS5 cohesin associated factor A (PDS5A), transcript variant 1, mRNA.
PGM5	5239	Homo sapiens phosphoglucomutase 5 (PGM5), mRNA.
PHF12	57649	Homo sapiens PHD finger protein 12 (PHF12), transcript variant 1, mRNA.
PHF19	26147	Homo sapiens PHD finger protein 19 (PHF19), transcript variant 2, mRNA.
PIK3AP1	118788	Homo sapiens phosphoinositide-3-kinase adaptor protein 1 (PIK3AP1), mRNA.
PITPNA	5306	Homo sapiens phosphatidylinositol transfer protein alpha (PITPNA), mRNA.

PLAG1	5324	Homo sapiens PLAG1 zinc finger (PLAG1), transcript variant 2, mRNA.
PLCB1	23236	Homo sapiens phospholipase C beta 1 (PLCB1), transcript variant 1, mRNA.
PLCH1	23007	Homo sapiens phospholipase C eta 1 (PLCH1), transcript variant 1, mRNA.
PLEKHA1	59338	Homo sapiens pleckstrin homology domain containing A1 (PLEKHA1), transcript variant 2, mRNA.
PLEKHH1	57475	Homo sapiens pleckstrin homology, MyTH4 and FERM domain containingH1 (PLEKHH1), mRNA.
POM121C	100101267	Homo sapiens POM121 transmembrane nucleoporin C (POM121C), transcript variant 1, mRNA.
POU2F1	5451	Homo sapiens POU class 2 homeobox 1 (POU2F1), transcript variant 2, mRNA.
PPM1L	151742	Homo sapiens protein phosphatase, Mg2+/Mn2+ dependent 1L (PPM1L), transcript variant 2, mRNA.
PPP1R12B	4660	Homo sapiens protein phosphatase 1 regulatory subunit 12B(PPP1R12B), transcript variant 5, mRNA.
PPTC7	160760	Homo sapiens protein phosphatase targeting COQ7 (PPTC7), transcript variant 1, mRNA.
PREX2	80243	Homo sapiens phosphatidylinositol-3,4,5-trisphosphate dependent Rac exchange factor 2 (PREX2), transcript variant 1, mRNA.
PRPS1	5631	Homo sapiens phosphoribosyl pyrophosphate synthetase 1 (PRPS1), transcript variant 2, mRNA.
PRRG1	5638	Homo sapiens proline rich and Gla domain 1 (PRRG1), transcript variant 1, mRNA.
PRTFDC1	56952	Homo sapiens phosphoribosyl transferase domain containing 1(PRTFDC1), transcript variant 2, mRNA.
PRUNE2	158471	Homo sapiens prune homolog 2 with BCH domain (PRUNE2), transcript variant 2, mRNA.
PTPRA	5786	Homo sapiens protein tyrosine phosphatase receptor type A (PTPRA), transcript variant 4, mRNA.
PURB	5814	Homo sapiens purine rich element binding protein B (PURB), mRNA.
RAP2C	57826	Homo sapiens RAP2C, member of RAS oncogene family (RAP2C), transcript variant 1, mRNA.
RASAL2	9462	Homo sapiens RAS protein activator like 2 (RASAL2), transcript variant 1, mRNA.
RBBP4	5928	Homo sapiens RB binding protein 4, chromatin remodeling factor (RBBP4), transcript variant 2, mRNA.
RIPK2	8767	Homo sapiens receptor interacting serine/threonine kinase 2(RIPK2), transcript variant 2, mRNA.
RMI1	80010	Homo sapiens RecQ mediated genome instability 1 (RMI1), transcript variant 1, mRNA.
RNF144A	9781	Homo sapiens ring finger protein 144A (RNF144A), transcript variant1, mRNA.
ROBO1	6091	Homo sapiens roundabout guidance receptor 1 (ROBO1), transcript variant 4, mRNA.
RORA	6095	Homo sapiens RAR related orphan receptor A (RORA), transcript variant 3, mRNA.
SDHC	6391	Homo sapiens succinate dehydrogenase complex subunit C (SDHC), transcript variant 2, mRNA; nuclear gene for mitochondrial product.
SDK1	221935	Homo sapiens sidekick cell adhesion molecule 1 (SDK1), transcript variant 2, mRNA.
SEC22C	9117	Homo sapiens SEC22 homolog C, vesicle trafficking protein (SEC22C), transcript variant 3, mRNA.
SEMA6A	57556	Homo sapiens semaphorin 6A (SEMA6A), transcript variant 1, mRNA.
SENP1	29843	Homo sapiens SUMO specific peptidase 1 (SENP1), transcript variant1, mRNA.
SERPIND1	3053	Homo sapiens serpin family D member 1 (SERPIND1), mRNA.
SGCD	6444	Homo sapiens sarcoglycan delta (SGCD), transcript variant 1, mRNA.
SGIP1	84251	Homo sapiens SH3GL interacting endocytic adaptor 1 (SGIP1), transcript variant 2, mRNA.
SH2D1A	4068	Homo sapiens SH2 domain containing 1A (SH2D1A), transcript variant2, mRNA.
SH3TC2	79628	Homo sapiens SH3 domain and tetratricopeptide repeats 2 (SH3TC2), mRNA.
SHANK1	50944	Homo sapiens SH3 and multiple ankyrin repeat domains 1 (SHANK1), mRNA.
SIAH3	283514	Homo sapiens siah E3 ubiquitin protein ligase family member 3(SIAH3), mRNA.
SKA2	348235	Homo sapiens spindle and kinetochore associated complex subunit 2(SKA2), transcript variant 2, mRNA.
SLAMF6	114836	Homo sapiens SLAM family member 6 (SLAMF6), transcript variant 1, mRNA.
SLC16A7	9194	Homo sapiens solute carrier family 16 member 7 (SLC16A7), transcript variant 2, mRNA.
SLC22A23	63027	Homo sapiens solute carrier family 22 member 23 (SLC22A23), transcript variant 3, mRNA.
SLC35E3	55508	Homo sapiens solute carrier family 35 member E3 (SLC35E3), transcript variant 2, mRNA.
SLC38A10	124565	Homo sapiens solute carrier family 38 member 10 (SLC38A10), transcript variant 1, mRNA.
SLC4A8	9498	Homo sapiens solute carrier family 4 member 8 (SLC4A8), transcript variant 1, mRNA.
SLTM	79811	Homo sapiens SAFB like transcription modulator (SLTM), transcript variant 2, mRNA.
SMC4	10051	Homo sapiens structural maintenance of chromosomes 4 (SMC4), transcript variant 2, mRNA.
SMURF2	64750	Homo sapiens SMAD specific E3 ubiquitin protein ligase 2 (SMURF2), mRNA.
SNRK	54861	Homo sapiens SNF related kinase (SNRK), transcript variant 2, mRNA.
SOD2	6648	Homo sapiens superoxide dismutase 2 (SOD2), transcript variant 1, mRNA; nuclear gene for mitochondrial product.
SORCS1	114815	Homo sapiens sortilin related VPS10 domain containing receptor 1(SORCS1), transcript variant 2, mRNA.
SP1	6667	Homo sapiens Sp1 transcription factor (SP1), transcript variant 3, mRNA.
SP2	6668	Homo sapiens Sp2 transcription factor (SP2), mRNA.
SPON1	10418	Homo sapiens spondin 1 (SPON1), mRNA.
SPTBN1	6711	Homo sapiens spectrin beta, non-erythrocytic 1 (SPTBN1), transcript variant 1, mRNA.
SRGAP1	57522	Homo sapiens SLIT-ROBO Rho GTPase activating protein 1 (SRGAP1), transcript variant 2, mRNA.
SRGAP2	23380	Homo sapiens SLIT-ROBO Rho GTPase activating protein 2 (SRGAP2), transcript variant 3, mRNA.
STK3	6788	Homo sapiens serine/threonine kinase 3 (STK3), transcript variant2, mRNA.
STX17	55014	Homo sapiens syntaxin 17 (STX17), mRNA.
STXBP5	134957	Homo sapiens syntaxin binding protein 5 (STXBP5), transcript variant 2, mRNA.
STXBP5L	9515	Homo sapiens syntaxin binding protein 5L (STXBP5L), transcript variant 2, mRNA.
SUMO2	6613	Homo sapiens small ubiquitin like modifier 2 (SUMO2), transcript variant 2, mRNA.
SYN2	6854	Homo sapiens synapsin II (SYN2), transcript variant IIb, mRNA.
SYNPO2	171024	Homo sapiens synaptopodin 2 (SYNPO2), transcript variant 2, mRNA.
SYT14	255928	Homo sapiens synaptotagmin 14 (SYT14), transcript variant 1, mRNA.

SYT4	6860	Homo sapiens synaptotagmin 4 (SYT4), mRNA.
TAL2	6887	Homo sapiens TAL bHLH transcription factor 2 (TAL2), mRNA.
TAOK1	57551	Homo sapiens TAO kinase 1 (TAOK1), transcript variant 1, mRNA.
TBC1D23	55773	Homo sapiens TBC1 domain family member 23 (TBC1D23), transcript variant 1, mRNA.
TBL2	26608	Homo sapiens transducin beta like 2 (TBL2), transcript variant 2, mRNA.
TBRG1	84897	Homo sapiens transforming growth factor beta regulator 1 (TBRG1), transcript variant 1, mRNA.
TCN1	6947	Homo sapiens transcobalamin 1 (TCN1), mRNA.
TEDDM1	127670	Homo sapiens transmembrane epididymal protein 1 (TEDDM1), mRNA.
THSD7A	221981	Homo sapiens thrombospondin type 1 domain containing 7A (THSD7A), mRNA.
TIAM2	26230	Homo sapiens TIAM Rac1 associated GEF 2 (TIAM2), transcript variant2, mRNA.
TJP1	7082	Homo sapiens tight junction protein 1 (TJP1), transcript variant 3, mRNA.
TLL2	7093	Homo sapiens tolloid like 2 (TLL2), mRNA.
TMCC1	23023	Homo sapiens transmembrane and coiled-coil domain family 1 (TMCC1), transcript variant 1, mRNA.
TMEM106B	54664	Homo sapiens transmembrane protein 106B (TMEM106B), transcript variant 2, mRNA.
TMEM132D	121256	Homo sapiens transmembrane protein 132D (TMEM132D), mRNA.
TMEM135	65084	Homo sapiens transmembrane protein 135 (TMEM135), transcript variant 2, mRNA.
TMEM184A	202915	Homo sapiens transmembrane protein 184A (TMEM184A), mRNA.
TMEM225	338661	Homo sapiens transmembrane protein 225 (TMEM225), transcript variant 1, mRNA.
TMEM33	55161	Homo sapiens transmembrane protein 33 (TMEM33), mRNA.
TMEM69	51249	Homo sapiens transmembrane protein 69 (TMEM69), mRNA.
TNIK	23043	Homo sapiens TRAF2 and NCK interacting kinase (TNIK), transcript variant 2, mRNA.
TNIP1	10318	Homo sapiens TNFAIP3 interacting protein 1 (TNIP1), transcript variant 1, mRNA.
TRDMT1	1787	Homo sapiens tRNA aspartic acid methyltransferase 1 (TRDMT1), transcript variant f, mRNA.
TRDN	10345	Homo sapiens triadin (TRDN), transcript variant 2, mRNA.
TRIM9	114088	Homo sapiens tripartite motif containing 9 (TRIM9), transcript variant 3, mRNA.
TRIML2	205860	Homo sapiens tripartite motif family like 2 (TRIML2), transcript variant 1, mRNA.
TRIP12	9320	Homo sapiens thyroid hormone receptor interactor 12 (TRIP12), transcript variant 1, mRNA.
TSC22D1	8848	Homo sapiens TSC22 domain family member 1 (TSC22D1), transcript variant 3, mRNA.
TSHZ1	10194	Homo sapiens teashirt zinc finger homeobox 1 (TSHZ1), transcript variant 1, mRNA.
TSTD2	158427	Homo sapiens thiosulfate sulfurtransferase like domain containing 2(TSTD2), mRNA.
TTC28	23331	Homo sapiens tetratricopeptide repeat domain 28 (TTC28), transcript variant 1, mRNA.
TTC29	83894	Homo sapiens tetratricopeptide repeat domain 29 (TTC29), transcript variant 1, mRNA.
TUBGCP3	10426	Homo sapiens tubulin gamma complex associated protein 3 (TUBGCP3), transcript variant 2, mRNA.
UHRF2	115426	Homo sapiens ubiquitin like with PHD and ring finger domains 2(UHRF2), transcript variant 1, mRNA.
UNC79	57578	Homo sapiens unc-79 homolog, NALCN channel complex subunit (UNC79), transcript variant 1, mRNA.
UNC80	285175	Homo sapiens unc-80 homolog, NALCN channel complex subunit (UNC80), transcript variant 3, mRNA.
UQCRC1	7384	Homo sapiens ubiquinol-cytochrome c reductase core protein 1(UQCRC1), mRNA; nuclear gene for mitochondrial product.
USP13	8975	Homo sapiens ubiquitin specific peptidase 13 (USP13), mRNA.
USP15	9958	Homo sapiens ubiquitin specific peptidase 15 (USP15), transcript variant 1, mRNA.
USP38	84640	Homo sapiens ubiquitin specific peptidase 38 (USP38), transcript variant 2, mRNA.
USP49	25862	Homo sapiens ubiquitin specific peptidase 49 (USP49), transcript variant 1, mRNA.
UTY	7404	Homo sapiens ubiquitously transcribed tetratricopeptide repeat containing, Y-linked (UTY), transcript variant 4, mRNA.
VEGFD	2277	Homo sapiens vascular endothelial growth factor D (VEGFD), mRNA.
VPS36	51028	Homo sapiens vacuolar protein sorting 36 homolog (VPS36), transcript variant 2, mRNA.
VPS45	11311	Homo sapiens vacuolar protein sorting 45 homolog (VPS45), transcript variant 2, mRNA.
VPS50	55610	Homo sapiens VPS50 subunit of EARP/GARPII complex (VPS50), transcript variant 3, mRNA.
WDR77	79084	Homo sapiens WD repeat domain 77 (WDR77), transcript variant 1, mRNA.
XDH	7498	Homo sapiens xanthine dehydrogenase (XDH), mRNA.
XIRP2	129446	Homo sapiens xin actin binding repeat containing 2 (XIRP2), transcript variant 2, mRNA.
ZDHHC5	25921	Homo sapiens zinc finger DHHC-type palmitoyltransferase 5 (ZDHHC5), mRNA.
ZFHX3	463	Homo sapiens zinc finger homeobox 3 (ZFHX3), transcript variant B, mRNA.
ZFP36L2	678	Homo sapiens ZFP36 ring finger protein like 2 (ZFP36L2), mRNA.
ZFP69B	65243	Homo sapiens ZFP69 zinc finger protein B (ZFP69B), transcript variant 2, mRNA.
ZKSCAN1	7586	Homo sapiens zinc finger with KRAB and SCAN domains 1 (ZKSCAN1), transcript variant 2, mRNA.
ZNF24	7572	Homo sapiens zinc finger protein 24 (ZNF24), transcript variant 2, mRNA.
ZNF292	23036	Homo sapiens zinc finger protein 292 (ZNF292), transcript variant2, mRNA.
ZNF569	148266	Homo sapiens zinc finger protein 569 (ZNF569), transcript variant2, mRNA.
ZNF652	22834	Homo sapiens zinc finger protein 652 (ZNF652), transcript variant1, mRNA.
ZNF704	619279	Homo sapiens zinc finger protein 704 (ZNF704), transcript variant1, mRNA.
ZNF737	100129842	Homo sapiens zinc finger protein 737 (ZNF737), mRNA.
ZNF764	92595	Homo sapiens zinc finger protein 764 (ZNF764), transcript variant2, mRNA.

Supplementary Table S2. GO and KEGG functional analysis of miR-1290 target genes.

GO cellular component	Count	P-Value	Genes
nucleoplasm	88	0.000047	KDM5A, ATF1, BNC2, ARID4B, RORA, SMC4, EME1, MAGOH, SUMO2, ENSA, KPNA6, BBX, SLC16A7, UTY, NCOA2, USP49, MBNL2, RMI1, DYRK1A, ETV3, CBFA2T3, SENP1, WDR77, FRMD7, SLTM, TNIK, PPP1R12B, DTL, KPNB1, USP13, KMT2D, FOXC1, CTDP1, NOTCH1, UHRF2, PLAG1, SYNPO2, CUL2, CAMK2A, ANKRD12, ARHGAP17, ZNF24, C1ORF52, PDS5A, NLK, TBRG1, PURB, NUAK1, CHTF8, ATXN1, RBBP4, DHX33, ERBB4, SRGAP2, MAPK6, LNPK, TRDMT1, ATF7IP, MAP3K2, ZFHX3, POU2F1, PLEKHA1, SMURF2, ERCC6L2, SIAH3, PHF12, ESRRB, CDC5L, ATP2B1, GRHL1, GTF2H3, A1CF, CUL4A, MOB1A, SP2, TRIML2, TNIP1, SP1, ERCC4, AGO1, CAMK4, HNRNPF, NAPEPLD, MDM4, TRIP12, BRWD1, PHF19, NEK11
cytoplasm	114	0.000049	PANK1, FBXO27, PITPNA, MSI2, ADARB2, IFIT1, FGF5, AKR7A2, MAGOH, CAPN2, KPNA6, GAS2L3, MBNL2, DYRK1A, ACSL4, WDR77, CLIP3, TNIK, KPNB1, CSNK1G1, GSTCD, ARHGAP17, NLK, KLC1, ZFP36L2, SKA2, STK3, LDHA, NUAK1, ATXN1, DHX33, MAP3K20, STXBP5, SRGAP2, CCDC141, SRGAP1, SPTBN1, ZFHX3, SMURF2, SIAH3, ESRRB, SH2D1A, DNAJC12, PARVA, A1CF, MOB1A, DLG1, CNOT7, SP1, AGO1, NAPEPLD, TUBGCP3, PLCH1, CPEB2, FGF12, MDM1, MSTN, FMN1, ARHGAP5, TTC29, BICD2, TTC28, ROBO1, PCMTD2, TRIM9, GRM5, SGCD, ENSA, KIF13B, HYDIN, NCOA2, USP49, TSC22D1, SGIP1, RIPK2, NAV1, IRAK3, CTTNBP2NL, SENP1, RNF144A, RAP2C, DCX, PPP1R12B, PLCB1, SHANK1, SEC22C, PRPS1, USP15, CTDP1, STX17, FAXC, PRUNE2, CAMK2A, NLRC3, NKD1, EGFR, MTHFSD, ABLIM1, FCHO2, LRRC8C, MAPK6, DYNC1I1, TRDMT1, MAP3K2, PLEKHA1, ERCC6L2, CDC5L, STXBP5L, TJP1, SNRK, TRIML2, CAMK4, TAOK1, NEK11
nucleus	112	0.003763	OTUD4, ATF1, PANK1, ZNF292, PITPNA, ARID4B, RORA, SMC4, EME1, MAGOH, CAPN2, KPNA6, FAM53C, ZNF569, BBX, VPS36, MAP3K7, DYRK1A, EBF1, ZFP69B, WDR77, CLIP3, ANKRD42, TNIK, KPNB1, CSNK1G1, KMT2D, NOTCH1, TSHZ1, GLIS3, ZNF24, PDS5A, NLK, LPP, ZFP36L2, STK3, LDHA, NUAK1, ATXN1, DHX33, ZNF704, MAP3K20, SPTBN1, ZKSCAN1, ZFHX3, POU2F1, FYB1, SMURF2, PHF12, ESRRB, PARVA, A1CF, MOB1A, DLG1, CNOT7, SP1, HNRNPF, AGO1, MDM4, BRWD1, CPEB2, FGF12, ZNF652, PHF19, MDM1, KDM5A, USP38, BNC2, FMN1, FHL5, SUMO2, ZNF764, NCOA2, TSC22D1, CTDSPL, IRAK3, ETV3, CBFA2T3, CDYL2, SENP1, SLTM, ELF5, PLCB1, DTL, CREB5, USP13, USP15, FOXC1, CTDP1, UHRF2, SYNPO2, CAMK2A, EGFR, TBRG1, PURB, RBBP4, ERBB4, MAPK6, DYNC1I1, ATF7IP, ERCC6L2, CDC5L, GRHL1, GTF2H3, SNRK, ERCC4, CAMK4, ASXL3, ZNF737, TRIP12, KCNK2, NEK11
glutamatergic synapse	14	0.004287	SYT4, NRP2, ATP2B1, SYN2, SLC4A8, GRM5, DLG1, LRRTM3, ERBB4, CAMK4, TNIK, PLCB1, SPTBN1, SHANK1
cytosol	104	0.005000	OTUD4, PANK1, FBXO27, PITPNA, ARID4B, MSI2, IFIT1, SMC4, PREX2, AKR7A2, CAPN2, KPNA6, BBX, VPS36, MAP3K7, ANK2, WDR77, TIAM2, CLIP3, CACNB4, FRMD7, TNIK, TBC1D23, KPNB1, CSNK1G1, NOTCH1, CUL2, ANKRD12, ARHGAP17, RASAL2, PDS5A, NLK, KLC1, LPP, SKA2, STK3, LDHA, ATXN1, MAP3K20, STXBP5, SRGAP2, SRGAP1, SPTBN1, MSR1, OSBPL6, FYB1, SMURF2, ESRRB, SH2D1A, PARVA, MOB1A, DLG1, CNOT7, HNRNPF, AGO1, TUBGCP3, PLCH1, TMCC1, BRWD1, MDM1, USP38, ARHGAP5, BICD2, KIF13B, BBS9, PGM5, SGIP1, RIPK2, DCC, TRDN, RAP2C, DCX, COL21A1, PPP1R12B, PLCB1, DTL, SHANK1, USP13, PRPS1, USP15, FOXC1, STX17, PLAG1, SYNPO2, CAMK2A, NLRC3, NRAS, RBBP4, ERBB4, VPS50, CHN2, FCHO2, MAPK6, XDH, DYNC1I1, ATF7IP, MAP3K2, PLEKHA1, TJP1, TRIML2, TNIP1, TAOK1, TRIP12, PIK3AP1
Schaffer collateral - CA1 synapse	6	0.008747	GRM5, PTPRA, DCC, STXBP5, SYN2, SHANK1
Z disc	7	0.015211	SYNPO2, XIRP2, PGM5, PARVA, ANK2, FHL5, PPP1R12B
recycling endosome	7	0.015739	SH3TC2, PANK1, VPS50, GPR161, ANK2, TNIK, DYNC1I1
axon	12	0.018175	UNC80, SYT4, NRP2, SEMA6A, CALCR, CNTN6, DCC, DYRK1A, KIF13B, GFRA1, OPRM1, ROBO1
astrocyte projection	3	0.018634	SYT4, GRM5, KCNK2
stress fiber	5	0.023077	ABLIM1, SYNPO2, XIRP2, PGM5, LPP
membrane raft	9	0.038186	CLIP3, CD4, DLG1, SMURF2, CAPN2, ANK2, ATP2B1, OPRM1, EGFR
cytoskeleton	15	0.038506	GAS2L3, SGIP1, RIPK2, DYRK1A, ANK2, TTC29, TRIM9, SGCD, FRMD7, FCHO2, KIF13B, DCX, TNIK, PPP1R12B, PLEKHH1
SNARE complex	4	0.040518	STX17, STXBP5, SYN2, STXBP5L
integral component of postsynaptic membrane	4	0.042581	NLGN3, GRM5, NRP2, OPRM1
receptor complex	8	0.047925	NOTCH1, FCRL5, ERBB4, PTPRA, IL5RA, GFRA1, EGFR, KCTD16

neuron projection	11	0.048318	SLC4A8, GRM5, DLG1, FRMD7, CAMK2A, DCX, ANK2, OPRM1, CCDC141, CPEB2, SHANK1
host cell	4	0.051347	POM121C, KPNA6, IFIT1, KPNB1
cytoplasmic side of plasma membrane	4	0.053665	DLG1, CACNB4, PGM5, ATP2B1
basolateral plasma membrane	8	0.066382	TJP1, ABCC4, DLG1, ATP7B, ERBB4, ANK2, ATP2B1, EGFR
integral component of presynaptic membrane	4	0.068592	ERBB4, NRXN1, ATP2B1, OPRM1
plasma membrane	88	0.072514	NRP2, MS4A3, ZDHHC5, IL5RA, FMN1, ARHGAP5, ANTXR2, BICD2, ROBO1, PREX2, UNC80, GLIPR1, GRM5, ATP7B, SGCD, CAPN2, SLC16A7, THSD7A, MAP3K7, MS4A10, ABCC4, SGIP1, DCC, ACSL4, ANK2, IRAK3, GFRA1, OPRM1, SYN2, TRDN, CLIP3, RNF144A, RAP2C, CACNB4, FRMD7, MMP16, KCNMA1, SHANK1, CSNK1G1, COLEC12, NLGN3, USP15, NOTCH1, STX17, NRXN1, ARHGAP17, PDS5A, LPP, NKD1, EGFR, PALM2AKAP2, NRAS, CALCR, SH3TC2, ERBB4, PRRG1, FCHO2, LRRC8C, SLAMF6, STXBP5, SRGAP2, CCDC141, SPTBN1, TMEM184A, MSR1, CD164, SYT4, OSBPL6, FYB1, PLEKHA1, SMURF2, CNTN6, PARVA, ATP2B1, STXBP5L, TJP1, SLC4A8, SDK1, CD4, DLG1, PTPRA, HNRNPF, FAT3, PLCH1, PIK3AP1, KCNK2, MUC21, UNC79
sarcolemma	5	0.072522	DLG1, SGCD, PGM5, ANK2, OPRM1
nuclear membrane	8	0.073179	OSBPL6, POM121C, NRXN1, PLCB1, DTL, EGFR, KPNB1, SENP1
membrane	48	0.077724	NRP2, STX17, LAMA3, ZDHHC5, IL5RA, KLC1, EGFR, PALM2AKAP2, GLIPR1, NRAS, LDHA, ATP7B, CHST11, VPS50, CHN2, LRRC8C, KPNA6, BBS9, MARCHF6, MSR1, SYT4, ABCC4, OSBPL6, PLEKHA1, HGF, ELMOD2, CDC5L, ACSL4, ANK2, AP3B1, VEGFD, SORCS1, ATP2B1, RAP2C, TIAM2, SLC4A8, DLG1, CD4, CNOT7, SLTM, CCPG1, PTPRA, HNRNPF, TMEM106B, TUBGCP3, PIK3AP1, KPNB1, SHANK1
endocytic vesicle membrane	4	0.094052	COLEC12, MSR1, GPR161, CAMK2A
postsynaptic membrane	6	0.094231	ERBB4, KCNMA1, ANK2, SRGAP2, SHANK1, KCTD16

GO molecular function	Count	P-Value	Genes
protein binding	227	0.000016	ATF1, SPON1, PITPNA, ZDHHC5, ANTXR2, IFIT1, SMC4, MAGOH, FAM53C, THSD7A, VPS36, TSTD2, EBF1, ANK2, WDR77, GPR161, UQCRC1, FREM1, KCTD16, ZNF24, NLK, SKA2, NUAK1, LDHA, DHX33, POM121C, ZNF704, SLAMF6, SPTBN1, MSR1, FYB1, SIAH3, SLC35E3, SH2D1A, PARVA, A1CF, TCN1, HNRNPF, AGO1, TMCC1, NRP2, MSTN, ARHGAP5, ROBO1, PCMTD2, TRIM9, GLIPR1, ENSA, KIF13B, BBS9, NXPE3, NCOA2, GRAMD2A, TSC22D1, FCRL5, RIPK2, RMI1, SORCS1, AP3B1, CBFA2T3, CDYL2, CTTNBP2NL, SLC22A23, RNF144A, RAP2C, TAL2, SLTM, TMEM33, KCNMA1, TMEM106B, PLCB1, FOXC1, CTDP1, STX17, SYNPO2, NKD1, EGFR, TMEM69, PURB, NRAS, CHN2, XDH, MARCHF6, DYNC1I1, MAP3K2, PLEKHA1, ERCC6L2, VEGFD, GRHL1, GTF2H3, STXBP5L, TJP1, TRIML2, KLHL6, TAOK1, TRIP12, NEK11, OTUD4, GALNT15, FBXO27, IL5RA, ARID4B, RORA, MSI2, AKR7A2, EME1, KPNA6, CAPN2, ZNF569, SLC16A7, MAP3K7, GAS2L3, SEMA6A, PPTC7, HGF, ZFP69B, DYRK1A, SDHC, CLIP3, CACNB4, FRMD7, TNIK, TBC1D23, KPNB1, CSNK1G1, KMT2D, NOTCH1, HTRA3, GSTCD, CUL2, GLIS3, ARHGAP17, RASAL2, PDS5A, KLC1, LPP, ZFP36L2, STK3, PALM2AKAP2, ATXN1, CALCR, FUT9, MAP3K20, SRGAP2, CCDC141, SRGAP1, ZKSCAN1, TMEM132D, CD164, SYT4, ZFHX3, OSBPL6, POU2F1, SMURF2, PHF12, DNAJC12, CYP4F11, MOB1A, DLG1, CNOT7, SP2, SP1, CCPG1, PTPRA, MDM4, TUBGCP3, FGF12, ZNF652, PHF19, MDM1, KDM5A, USP38, MS4A3, BNC2, CXORF66, FHL5, BICD2, GRM5, ATP7B, SUMO2, ZNF764, ABCC4, USP49, DCC, SGIP1, CTDSPL, IRAK3, OPRM1, NIPSNAP2, TRDN, SENP1, ELF5, DCX, PPP1R12B, DTL, PRTFDC1, SHANK1, CREB5, SEC22C, NLGN3, USP13, PRPS1, USP15, UHRF2, NRXN1, CAMK2A, NLRC3, TBRG1, ABLIM1, RBBP4, ERBB4, VPS50, PRRG1, FCHO2, LRRC8C, MAPK6, ATF7IP, NDUFA5, CDC5L, ATP2B1, SOD2, CUL4A, CD4, TNIP1, ERCC4, VPS45, PIK3AP1
protein kinase activity	17	0.000263	MAP3K2, RIPK2, DYRK1A, MMD2, NLK, EGFR, STK3, NUAK1, SNRK, SLTM, CAMK4, TAOK1, MAP3K20, TNIK, MAP3K7, NEK11, CSNK1G1
protein serine/threonine/tyrosine kinase activity	18	0.000404	MAP3K2, RIPK2, CAMK2A, DYRK1A, NLK, EGFR, STK3, NUAK1, SNRK, ERBB4, CAMK4, TAOK1, MAP3K20, TNIK, MAPK6, MAP3K7, NEK11, CSNK1G1
metal ion binding	62	0.000680	SPON1, NRP2, BNC2, GALNT15, ZNF292, FHL5, ANTXR2, ADARB2, GALNT10, ATP7B, EME1, ZNF569, UTY, ZNF764, MBNL2, PPTC7, EBF1, ZFP69B, CTDSPL, SDHC, CBFA2T3, RNF144A, KCNMA1, UQCRC1, FREM1, CREB5, COLEC12, KMT2D, PPM1L, UHRF2, PLAG1, NRXN1, TSHZ1, PRUNE2, CAMK2A, GLIS3, ZNF24, LPP, ZFP36L2, ABLIM1, NUAK1, ZNF704, CHN2, XIRP2, ZKSCAN1, LNPK, MAP3K2, SIAH3, PHF12, ATP2B1,

			GTF2H3, SOD2, MOB1A, CNOT7, SP2, SP1, ASXL3, ZNF737, MDM4, ZNF652, PHF19, NEK11
protein serine/threonine kinase activity	16	0.001209	MAP3K2, RIPK2, CAMK2A, DYRK1A, IRAK3, NLK, STK3, NUAK1, SNRK, TAOK1, MAP3K20, TNIK, MAPK6, MAP3K7, NEK11, CSNK1G1
identical protein binding	42	0.002943	ATF1, PRPS1, USP15, NRP2, NOTCH1, MSTN, HTRA3, CAMK2A, MSI2, ZNF24, EGFR, STK3, ROBO1, GRM5, LDHA, ATXN1, FCHO2, SRGAP2, MAP3K7, LNPK, POU2F1, SMURF2, RIPK2, HGF, DYRK1A, CDC5L, VEGFD, SOD2, SYN2, SDK1, CD4, TNIP1, TRIML2, SP1, ERCC4, KCNMA1, NAPEPLD, TMCC1, PLCB1, PIK3AP1, SHANK1, CREB5
GTPase activator activity	12	0.004968	PREX2, TIAM2, ELMOD2, CHN2, ARHGAP17, STXBP5, RASAL2, PLCB1, SRGAP2, ARHGAP5, SRGAP1, STXBP5L
MAP kinase kinase kinase activity	4	0.005810	MAP3K2, MAP3K20, MAP3K7, EGFR
protein binding, bridging	6	0.021845	CUL4A, SYNPO2, CUL2, ANK2, DTL, TRDN
MAP kinase activity	3	0.021876	NLK, MAPK6, MAP3K7
protein kinase binding	15	0.023415	MAP3K2, STX17, ERCC6L2, CDC5L, IRAK3, ANK2, EGFR, DLG1, CD4, TBL2, TRIML2, KIF13B, DCX, PPP1R12B, MAPK6
magnesium ion binding	9	0.026419	PRPS1, SNRK, MAP3K20, PGM5, IRAK3, NLK, MAP3K7, PRTFDC1, STK3
transcription regulatory region sequence-specific DNA binding	9	0.030746	KDM5A, KMT2D, FOXC1, ZFHX3, BNC2, ERBB4, SP1, ARID4B, GRHL1
cytoskeletal adaptor activity	3	0.034217	GAS2L3, ANK2, BICD2
single-stranded RNA binding	4	0.042006	HNRNPF, AGO1, ADARB2, A1CF
protein C-terminus binding	8	0.042342	TJP1, DLG1, ATXN1, ERCC4, SP1, OPRM1, VPS36, SHANK1
receptor binding	12	0.047761	BMP3, CD4, FYB1, MSTN, RIPK2, NRXN1, HGF, LAMA3, ENSA, GFRA1, PIK3AP1, TRDN
microtubule binding	9	0.053361	CLIP3, GAS2L3, SGIP1, KIF13B, DCX, FMN1, DYNC1I1, MDM1, SKA2
RNA polymerase II regulatory region sequence-specific DNA binding	11	0.058655	ATF1, PURB, FOXC1, SP1, GLIS3, ZFP69B, BBX, CDC5L, RORA, ZNF764, ETV3
RNA polymerase II transcription factor activity, sequence-specific DNA binding	28	0.058730	ATF1, FOXC1, PLAG1, TSHZ1, ZNF292, GLIS3, RORA, ZNF24, PURB, BBX, ZNF569, ZNF764, ZKSCAN1, ZFHX3, POU2F1, ESRRB, EBF1, ZFP69B, CDC5L, ETV3, GRHL1, SP2, TAL2, SP1, ZNF737, ELF5, ZNF652, CREB5
nuclear localization sequence binding	3	0.064829	POM121C, KPNA6, KPNB1
sequence-specific DNA binding	10	0.070271	ATF1, FOXC1, POU2F1, SLTM, SP1, ELF5, ESRRB, RORA, GRHL1, ZNF24
transmembrane signaling receptor activity	7	0.072245	CD4, NOTCH1, FCRL5, DCC, NRXN1, ANTXR2, EGFR
enzyme binding	11	0.072768	CD4, ZFHX3, NOTCH1, CAPN2, MDM4, ANK2, PLCB1, SOD2, EGFR, MARCHF6, KPNB1
ATP binding	32	0.078070	PRPS1, PANK1, CAMK2A, NLRC3, NLK, SMC4, EGFR, STK3, NUAK1, ATP7B, DHX33, ERBB4, MAP3K20, KIF13B, MAPK6, MAP3K7, MAP3K2, ABCC4, ERCC6L2, RIPK2, DYRK1A, ACSL4, IRAK3, ATP2B1, SYN2, SNRK, SLTM, TAOK1, CAMK4, TNIK, NEK11, CSNK1G1
SNARE binding	4	0.082195	TRIM9, SYT4, STX17, VPS50
rDNA binding	2	0.089205	DHX33, BNC2
ubiquitin-protein transferase activity	8	0.091850	CUL4A, RNF144A, UHRF2, SMURF2, CUL2, TRIP12, DTL, MARCHF6
ubiquitin conjugating enzyme binding	3	0.091887	RNF144A, SIAH3, MARCHF6
chromatin DNA binding	4	0.094116	KDM5A, NOTCH1, UTY, GRHL1

**GO biological process**	**Count**	**P-Value**	**Genes**
MAPK cascade	20	0.000000	MAP3K2, PPM1L, RIPK2, HGF, CAMK2A, IL5RA, GFRA1, RASAL2, NLK, EGFR, FGF5, DLG1, NRAS, ERBB4, PTPRA, TAOK1, TNIK, MAPK6, MAP3K7, SPTBN1
axon guidance	12	0.001042	NRP2, NOTCH1, SEMA6A, CNTN6, PTPRA, DCC, NRXN1, LAMA3, GFRA1, CCDC141, SPTBN1, ROBO1
positive regulation of protein localization to plasma membrane	6	0.001914	CLIP3, DLG1, NRXN1, ZDHHC5, EGFR, SPTBN1
Wnt signaling pathway, calcium modulating pathway	5	0.002382	AGO1, CAMK2A, PLCB1, NLK, MAP3K7
protein stabilization	10	0.003800	TBRG1, ATF7IP, USP13, MDM4, PARVA, ANK2, NLK, COA8, A1CF, STK3

protein phosphorylation	17	0.005059	MAP3K2, CAMK2A, DYRK1A, IRAK3, MMD2, NLK, STK3, NUAK1, GRM5, SNRK, PTPRA, CAMK4, TAOK1, MAP3K20, TNIK, MAPK6, NEK11
protein autophosphorylation	9	0.006476	ERBB4, CAMK4, TAOK1, CAMK2A, DYRK1A, IRAK3, TNIK, NLK, EGFR
T cell activation	5	0.007628	CD4, DLG1, KIF13B, SLAMF6, NLRC3
negative regulation of calcium ion-dependent exocytosis	3	0.007821	TRIM9, SYT4, NOTCH1
regulation of transcription, DNA-templated	26	0.007916	USP13, KMT2D, FOXC1, NOTCH1, BNC2, PLAG1, RORA, ARID4B, NLK, GRM5, ATXN1, POM121C, RBBP4, ZNF569, ZNF764, ZKSCAN1, ATF7IP, NCOA2, ZFHX3, PHF12, ESRRB, GTF2H3, SP1, ZNF737, ASXL3, PHF19
intracellular signal transduction	15	0.008814	NLRC3, IRAK3, NLK, STK3, PREX2, NUAK1, SNRK, CHN2, CAMK4, TAOK1, DCX, TNIK, PLCB1, MAPK6, NEK11
positive regulation of synaptic transmission, glutamatergic	4	0.009650	NLGN3, NRXN1, EGFR, SHANK1
positive regulation of transcription from RNA polymerase II promoter	30	0.009870	ATF1, KMT2D, FOXC1, NOTCH1, PLAG1, ZNF292, GLIS3, RORA, ARID4B, FHL5, ZNF24, EGFR, KPNA6, NCOA2, ZFHX3, POU2F1, RIPK2, HGF, ESRRB, CDC5L, AP3B1, GRHL1, SENP1, CNOT7, TNIP1, SP1, CCPG1, AGO1, ASXL3, MDM4
cellular response to granulocyte macrophage colony-stimulating factor stimulus	3	0.011711	CD4, ETV3, ZFP36L2
regulation of NMDA receptor activity	4	0.012667	DLG1, NRXN1, CAMK2A, OPRM1
positive regulation of protein phosphorylation	9	0.014141	FGF5, CLIP3, MOB1A, CD4, ERBB4, HGF, VEGFD, TNIK, EGFR
learning	5	0.014152	NLGN3, GRM5, ATXN1, NRXN1, PLCB1
positive regulation of transcription, DNA-templated	19	0.014678	KDM5A, ATF7IP, MAP3K2, ZFHX3, FOXC1, NOTCH1, MSTN, ESRRB, DYRK1A, RORA, EGFR, WDR77, RAP2C, CD4, SP1, ERBB4, CAMK4, PLCB1, CREB5
protein import into nucleus	6	0.016002	NOTCH1, POM121C, KPNA6, FAM53C, KPNB1, STK3
nervous system development	13	0.016199	PRPS1, SEMA6A, DYRK1A, NAV1, GFRA1, ROBO1, FGF5, ATXN1, FRMD7, ERBB4, DCX, SRGAP2, FGF12
dendritic spine development	3	0.016277	CAMK2A, ACSL4, SRGAP2
positive regulation of interleukin-2 production	4	0.017438	CD4, RIPK2, MAP3K7, SPTBN1
vascular endothelial growth factor signaling pathway	3	0.024299	FOXC1, NRP2, VEGFD
negative regulation of stem cell differentiation	3	0.024299	NOTCH1, ESRRB, ZFP36L2
vocalization behavior	3	0.024299	NLGN3, NRXN1, SHANK1
activation of JUN kinase activity	4	0.024627	MAP3K2, RIPK2, TAOK1, MAP3K20
negative regulation of skeletal muscle tissue growth	2	0.030363	MSTN, TLL2
response to magnetism	2	0.030363	TJP1, SOD2
resolution of meiotic recombination intermediates	3	0.030369	EME1, ERCC4, RMI1
positive regulation of protein kinase B signaling	8	0.030375	FGF5, CALCR, ERBB4, NRXN1, HGF, PIK3AP1, EGFR, STK3
activation of protein kinase activity	5	0.030412	MOB1A, DLG1, TAOK1, TNIK, STK3
regulation of transcription from RNA polymerase II promoter	37	0.031918	ATF1, FOXC1, TSHZ1, ZNF292, GLIS3, RORA, ARID4B, ZNF24, EGFR, PURB, ZNF704, BBX, ZNF569, ZNF764, ZKSCAN1, ZFHX3, POU2F1, TSC22D1, ESRRB, EBF1, ZFP69B, CDC5L, ETV3, GRHL1, SOD2, WDR77, SLTM, SP2, TNIP1, TAL2, SP1, ZNF737, ELF5, MDM4, BRWD1, ZNF652, CREB5
cellular response to epidermal growth factor stimulus	4	0.035053	FOXC1, ERBB4, EGFR, ZFP36L2
interleukin-12-mediated signaling pathway	4	0.035053	HNRNPF, PITPNA, PLCB1, SOD2
regulation of small GTPase mediated signal transduction	7	0.035778	PREX2, TIAM2, CHN2, ARHGAP17, SRGAP2, ARHGAP5, SRGAP1
stress-activated protein kinase signaling cascade	3	0.036968	TAOK1, TNIK, STK3
ubiquitin-dependent protein catabolic process	10	0.038060	CUL4A, RNF144A, USP13, USP15, USP38, USP49, SMURF2, CUL2, TRIP12, DTL

nucleotide-excision repair, DNA incision, 3'-to lesion	3	0.040455	CUL4A, ERCC4, GTF2H3
nucleotide-excision repair, preincision complex stabilization	3	0.040455	CUL4A, ERCC4, GTF2H3
multicellular organism growth	5	0.044520	TAL2, PLEKHA1, SP2, RMI1, PLAG1
secretory columnal luminar epithelial cell differentiation involved in prostate glandular acinus development	2	0.045198	NOTCH1, WDR77
positive regulation of NF-kappaB transcription factor activity	7	0.049730	TRIML2, DHX33, RIPK2, CAMK2A, ANKRD42, IRAK3, MAP3K7
cell-cell junction organization	3	0.051610	TJP1, SDK1, XIRP2
social behavior	4	0.051936	NLGN3, ATXN1, NRXN1, SHANK1
protein deubiquitination	9	0.053383	OTUD4, USP13, USP15, USP38, USP49, TNIP1, SMURF2, MDM4, MAP3K7
positive regulation of JNK cascade	5	0.058036	RIPK2, TAOK1, TNIK, PLCB1, STK3
global genome nucleotide-excision repair	3	0.059582	CUL4A, ERCC4, GTF2H3
endocardium development	2	0.059806	NOTCH1, STK3
histone H2B conserved C-terminal lysine deubiquitination	2	0.059806	USP15, USP49
positive regulation of ERK1 and ERK2 cascade	8	0.061366	CD4, NOTCH1, CALCR, ERBB4, RIPK2, NRXN1, OPRM1, EGFR
positive regulation of excitatory postsynaptic potential	3	0.063717	NLGN3, NRXN1, SHANK1
negative regulation of myoblast differentiation	3	0.063717	ZFHX3, NOTCH1, MSTN
outflow tract septum morphogenesis	3	0.063717	NRP2, PARVA, ROBO1
regulation of cytoskeleton organization	3	0.063717	TJP1, TAOK1, CAPN2
hippo signaling	3	0.067945	TJP1, MOB1A, STK3
cytoskeleton organization	6	0.068946	ABLIM1, SEMA6A, MAP3K20, ANK2, TNIK, BRWD1
hemopoiesis	4	0.069356	CUL4A, CD164, SOD2, ZFP36L2
positive regulation of calcium ion transport	3	0.072263	CAMK2A, ANK2, ATP2B1
toll-like receptor 2 signaling pathway	2	0.074192	RIPK2, PIK3AP1
cell-cell adhesion	7	0.074614	TJP1, DLG1, DCC, LAMA3, FAT3, LPP, EGFR
neuron projection morphogenesis	4	0.074759	TAOK1, TNIK, SRGAP2, EGFR
JNK cascade	4	0.074759	RIPK2, MAP3K20, FGF12, MAP3K7
positive regulation of viral genome replication	3	0.076668	NOTCH1, CNOT7, IFIT1
adult behavior	3	0.076668	NLGN3, NRXN1, SHANK1
calcium ion regulated exocytosis	3	0.076668	SYT4, ARHGAP17, SYN2
negative regulation of protein kinase B signaling	4	0.077529	DLG1, MSTN, PLEKHA1, XDH
synapse assembly	4	0.080344	NLGN3, SDK1, ERBB4, NRXN1
extracellular matrix disassembly	4	0.083202	MMP16, CAPN2, TLL2, KLK7
post-embryonic development	4	0.083202	CHST11, TAL2, PLEKHA1, SOD2
regulation of catalytic activity	11	0.084298	PREX2, MMP16, FRMD7, AP3B1, ARHGAP17, STXBP5, PLCB1, SOD2, ARHGAP5, SRGAP1, STXBP5L
sprouting angiogenesis	3	0.085720	NOTCH1, VEGFD, PARVA
intrinsic apoptotic signaling pathway	3	0.085720	CUL4A, CUL2, COA8
mesenchymal cell development	2	0.088359	FOXC1, NOTCH1
circadian regulation of gene expression	4	0.089047	KDM5A, NCOA2, ZFHX3, RORA

protein dephosphorylation	6	0.089931	DLG1, CTDP1, PPM1L, PTPRA, CTDSPL, CTTNBP2NL
protein localization to plasma membrane	6	0.089931	DLG1, FYB1, FCHO2, ANK2, TNIK, SPTBN1
MyD88-dependent toll-like receptor signaling pathway	3	0.090362	TNIP1, IRAK3, MAP3K7
regulation of MAPK cascade	3	0.090362	TAOK1, TNIK, STK3
negative regulation of cell proliferation	12	0.093901	TBRG1, CD164, NOTCH1, CNOT7, RBBP4, ERBB4, MDM4, OPRM1, ETV3, SOD2, CBFA2T3, STK3
fat cell differentiation	4	0.095060	CLIP3, LRRC8C, BBS9, PLCB1
protein K48-linked deubiquitination	3	0.095075	OTUD4, USP13, USP15
cell cycle	10	0.099845	TBRG1, UHRF2, RBBP4, CCPG1, ENSA, MAP3K20, CDC5L, MAPK6, SMC4, TTC28

**KEGG Pathway**	**Count**	**P-Value**	**Genes**
MAPK signaling pathway	13	0.004391	MAP3K2, HGF, VEGFD, NLK, EGFR, STK3, FGF5, NRAS, CACNB4, ERBB4, TAOK1, MAP3K20, MAP3K7
Calcium signaling pathway	11	0.007918	FGF5, GRM5, ERBB4, HGF, CAMK4, CAMK2A, VEGFD, ATP2B1, PLCB1, EGFR, TRDN
Gap junction	6	0.017113	TJP1, MAP3K2, GRM5, NRAS, PLCB1, EGFR
Aldosterone synthesis and secretion	6	0.025944	ATF1, CAMK4, CAMK2A, ATP2B1, PLCB1, CREB5
Long-term potentiation	5	0.027302	GRM5, NRAS, CAMK4, CAMK2A, PLCB1
Estrogen signaling pathway	7	0.030617	NCOA2, NRAS, SP1, OPRM1, PLCB1, EGFR, CREB5
Hippo signaling pathway - fly	5	0.031418	MOB1A, DLG1, TSHZ1, LPP, STK3
Axon guidance	8	0.036152	NRAS, ABLIM1, SEMA6A, DCC, CAMK2A, SRGAP2, SRGAP1, ROBO1
Nucleocytoplasmic transport	6	0.037267	POM121C, TMEM33, MAGOH, SUMO2, KPNA6, KPNB1
Oxytocin signaling pathway	7	0.048120	NRAS, CACNB4, CAMK4, CAMK2A, PPP1R12B, PLCB1, EGFR
Cushing syndrome	7	0.049383	KMT2D, SP1, CAMK2A, PLCB1, KCNK2, EGFR, CREB5
Focal adhesion	8	0.056365	HGF, LAMA3, CAPN2, VEGFD, PARVA, PPP1R12B, ARHGAP5, EGFR
GnRH signaling pathway	5	0.074504	MAP3K2, NRAS, CAMK2A, PLCB1, EGFR
Dorso-ventral axis formation	3	0.077056	NOTCH1, CPEB2, EGFR
Cortisol synthesis and secretion	4	0.099392	SP1, PLCB1, KCNK2, CREB5

Supplementary Table S3. The expression of miR-1290 in different cancers and its biological effect.

miR-1290				
**Type of cancer**	**Expression**	**Source/Experimental models**	**Biological effect of miR-1290**	**Reference**
**Colorectal cancer**	Overexpression	SW480 and HT-29 colorectal cancer (CRC) cells and tissues	miR-1290 initiates and promotes progression of the tumor by targeting 3′ UTRs of *INPP4B*, a negative regulator of PI3K/Akt signaling pathway; miR-1290 promotes proliferation of CRC cells by inhibiting expression of cyclin-dependent kinase inhibitor (p27) and increasing expression of cyclin D1	[145,146]
	Overexpression	CRC tissues, SW620 cell line	miR-1290 activates pro-survival pathways such as Akt and NF-κB to maintain proliferation of CRC cells; miR-1290 impairs cytokinesis by targeting *KIF13B* that generates multinucleated cells; Activation of Wnt and NF-κB pathways promotes transcription of miR-1290 and maintains CRC cell proliferation; Upregulation of reprogramming-related transcription factors such as c-Myc and Nanog in miR-1290-expressing cells	[150]
	Overexpression	CRC tissues, SW480-5FU$_{res}$, HCT116-5FU$_{res}$ cell lines, *in vivo* CRC xenografts mouse model	miR-1290 inhibits expression of *hMSH2*; miR-1290 reduces the sensitivity of CRC cells to 5-FU *in vitro;* miR-1290 is positively correlated with dMMR status and predicts poor prognosis in patients with stage II and III CRC who received 5-FU chemotherapy	[123]
	Downregulation	SW480-5FU$_{res}$ cell line; Murine tumor model	miR-1290 increases sensitivity to 5-FU treatment by direct targeting of hMSH2 and induces apoptosis *in vitro* and *in vivo*	
	Overexpression	Caco-2 cell line	Overexpression of miR-1290 in KRAS-wild type CRC cell line sensitive to sorafenib inhibits cell growth through S-phase cell cycle arrest	[154]
	Overexpression	Primary CRC and colorectal liver metastasis	miR-1290 is upregulated in liver metastasis in comparison to primary CRC; *In silico* prediction shows different targets for miR-1290 for example *AXL* involved in cell growth, invasion, metastasis, EMT, angiogenesis, drug resistance and stem cell maintenance; *CFLAR* encoding an important regulatory protein in extrinsic apoptotic pathway; *GAS7* responsible for maintaining microtubule stability and polarization, or *TMX4* acting as endoplasmic reductase required in protein folding and degradation pathways	[156–160]
	Overexpression	Plasma, CRC tissues	miR-1290 is overexpressed in CRC tissues in comparison to normal mucosa; Circulating miR-1290 is a novel promising biomarker for the early detection of CRC	[124]
	Overexpression	Serum-derived exosomes	miR-1290 is useful in the differentiation of patients at stage I CRC from healthy controls	[125]
	Overexpression	Serum and tissue specimens; LoVo and HT29 cell lines	Serum miR-1290 shows a potential to separate healthy individuals from patients with neoplasia (CRC and adenoma); Serum miR-1290 is an independent prognostic biomarker for predicting poorer overall survival (OS) in CRC patients and a predictor of tumor recurrence after surgery; miR-1290 is upregulated in CRC and adenoma tissues in comparison to healthy individuals; CRC cell lines overexpress miR-1290 and secrete it into the culture medium; miR-1290 contributes to the proliferation, migration and invasion *in vitro*	[126]
**Pancreatic cancer**	Overexpression	PanIN-1, PanIN-3 and pancreatic ductal adenocarcinoma (PDAC) tissues; Plasma; AsPC-1 and PANC-1 cells; Murine tumor model	Significant changes in miR-1290 expression in PanIN-3 and PDAC tissues in comparison to PanIN-1 confirms its involvement at early stage of pancreatic carcinogenesis; Circulating miR-1290 has potential diagnostic value for PDAC; High plasma miR-1290 is correlated with histological differentiation, pT stage, lymph node metastasis; miR-1290 promotes proliferation, migration and invasion *in vitro* and *in vivo*; 3′UTR of *IKK1* is a direct target for miR-1290	[167]
	Overexpression	Blood	High levels of miR-1290 predict poor OS in pancreatic cancer patients	[170]

	Overexpression	Plasma	High miR-1290 expression in older and male PDAC patients in comparison to healthy controls	[171]
	Overexpression	Serum	miR-1290 in combination with CA 19-9 improves diagnostic accuracy of PDAC	[129]
	Overexpression	Exosomes derived from Panc-1 and SUIT-2 cell lines; Human primary pancreatic stellate cells (PSCs) isolated from pancreatic cancer tissues	Exosomes containing miR-1290 induce *ACTA2* and fibrosis-related genes in PSCs which favors tumorigenesis; Exosomes increase proliferation and migration of PSCs	[172]
**Gastric cancer**	Overexpression	Serum-derived exosomes; SGC 7901 and AGS cell lines	Enhanced proliferation, migration and invasion of gastric cancer (GC) cells observed after incubation with exosomes rich in miR-1290 via targeting *NKD1*	[185]
	Overexpression	Human tissue specimens; Tumor-derived extracellular vesicles; MGC-803, GCL-1 and MFC cell lines; Murine tumor model	miR-1290 in extracellular vesicles secreted from GC cells contributes to immune escape through Grlh2/ZEB1/PDL-1 axis resulting in immune escape *in vitro* and *in vivo*	[187]
**Liver cancer**	Overexpression	Serum-derived exosomes; Tumor tissues; HUVEC, HepG2 and SMMC-7221 cell lines	miR-1290 is highly expressed in serum-derived exosomes from hepatocellular carcinoma (HCC) patients, tumor tissues, and HCC cell lines; *SMEK1* is a direct target of miR-1290 in HUVEC cell line; miR-1290 exerts proangiogenic properties *in vitro* and *in vivo*; miR-1290 is determined as a potential target of HCC therapy	[196]
	Overexpression	Plasma	Upregulation of circulating miR-1290 observed in recurrent recipients after liver transplantation, predicts poor OS and disease-free survival (DFS)	[199]
	Downregulation	HepG2 cells	Paclitaxel reduces expression of miR-1290; miR-1290 inhibits proliferation and enhances apoptosis *in vitro;* Putative targets for miR-1290 *(RAS, PI3K, PTEN, ECM, ITGB, c-myb)* are involved in PI3K/Akt signaling; Manipulation of miRNA level may serve as an important approach in paclitaxel therapy	[201]
**Esophageal cancer**	Overexpression	Tumor tissues	miR-1290 is upregulated in esophageal squamous cell carcinoma (ESCC) tissues compared to noncancerous esophageal mucosa; Overexpression of miR-1290 and low level of its target *NFIX* are significantly associated with positive lymph node metastasis, advanced tumor stage, short OS and DFS of ESCC patients	[130]
	Overexpression	KYSE410 and ECA 109 cells	Overexpression of miR-1290 significantly downregulates *NFIX* levels which promotes proliferation, migration and invasion *in vitro*	[212]
	Overexpression	Tumor tissues; ECA 109 and TE13 cells	miR-1290 overexpressed in ESCC tissues in comparison to adjacent normal mucosa is associated with tumor differentiation, N classification, and tumor-node metastasis stage in ESCC patients; miR-1290 enhances proliferation, migration, invasion, and downregulates expression of suppressor of cancer cell invasion (SCAI) *in vitro*	[213]
	Overexpression	Serum	High serum miR-1290 levels may discriminate ESCC patients from healthy controls and reflect progression of ESCC	[214]
**Lung cancer**	Overexpression	Tissue	miR-1290 is overexpressed in small-cell lung cancer (SCLC) tissues in comparison to normal lung tissue; miR-1290 is overexpressed in non-small-cell lung cancer (NSCLC) tissues in comparison to normal adjacent tissues	[218,219]
	Overexpression	Tissue, serum; A549, cell line; Murine tumor model	miR-1290 is a crucial driver for tumor initiation, progression and metastasis; *MTIG* is a direct target for miR-1290; Serum miR-1290 levels increased in stage I-III NSCLC patients compared with healthy individuals; Circulating miR-1290 corresponds with the response of patients ongoing EGFR tyrosine kinase inhibitor (TKI) treatment, and in some instances followed by either radio- or chemotherapy; Inhibition of miR-1290 with administration of locked nucleic acids (LNA) arrests the	[220]

			growth of tumor *in vivo*; miR-1290 is clinically useful as biomarker of disease progression and as a therapeutic target	
	Downregulation	A549 and HCC1588 cells	Inhibition of miR-1290 decreases stemness and EMT markers in NSCLC and suppresses proliferation, sphere-formation, colony formation and invasion of NSCLC	[221]
	Downregulation	Murine tumor model	AntagomiR-1290 suppresses NSCLC volume and weight initiated by CD133-positive cells *in vivo;* AntagomiR-1290 inhibits proliferation, clonogenicity, invasion and migration of CD13-positive cells by targeting fyn-related Src family tyrosine kinase	[222]
	Overexpression Downregulation	Tissues; A549 and H460 cell lines; Murine tumor model	Upregulation of miR-1290 in NSCLC tissues in comparison to normal adjacent tissue; Upregulation of miR-1290 in NSCLC cells; High miR-1290 levels are closely related to negative lymph node metastasis and advanced clinical stage (stage III); miR-1290 targets IRF2 and stimulates proliferation, colony formation, and invasion; miR-1290 enhances expression of cell cycle proteins (CDK2, CDK4) and stimulates EMT by upregulating N-cadherin and downregulating E-cadherin; Antagomir-1290 reduces tumor size and growth volume *in vivo*	[223]
	Overexpression	Tissues; A549, SPC-A1; Murine tumor model	miR-1290 promotes proliferation, cell cycle progression, invasion and inhibits apoptosis in lung adenocarcinoma (LADC) cells; miR-1290 promotes tumor growth, invasion and metastasis *in vivo;* miR-1290 targets *SOCS4* and activates JAK/STAT3 and PI3K/Akt signaling pathways; *SOCS4* is downregulated in LADC tissues in comparison to matched noncancerous lung tissues and correlates with poor clinical outcome	[224]
	Downregulation	A549 cells	*Polygonatum odoratum* lectin induces autophagy and apoptosis by downregulation of miR-1290 through inactivation of Wnt/ß-catenin pathway; *GSK3ß* a direct downstream target of miR-1290 is upregulated	[225]
	Overexpression	A549 cells	miR-1290 is a regulator of asiatic acid-induced apoptosis by targeting *BCL2* mRNA *in vitro*	[226]
	Overexpression	Tissues and serum	miR-1290 is upregulated in NSCLC tissues in comparison to non-tumor adjacent tissues; miR-1290 is positively correlated with high tumor stage and lymph node metastasis; miR-1290 levels are higher in NSCLC patients than in those with benign lung disease and healthy controls; miR-1290 is upregulated in serum of NSCLC patients with high TNM stage and positive lymph node metastasis; High serum miR-1290 levels predict poor survival	[131]
	Overexpression	Serum exosomes	miR-1290 is significantly higher in LADC patients than in healthy controls and it is associated with tumor stage, tumor size, lymph node metastasis and distant metastasis; Diagnostic efficacy of miR-1290 is higher than CEA, CYFRA21-1 and NSE; LADC patients with high miR-1290 levels have poorer progression-free survival (PFS) than those with low miR-1290 levels	[132]
	Overexpression	Tissues	miR-1290 is a predictive biomarker for response to platinum-based chemotherapy in advanced NSCLC	[229]
	Overexpression	Tissues	miR-1290 predicts response to platinum-based doublet chemotherapy in patients with LADC recurring after initial surgical resection	[230]
**Breast cancer**	Overexpression	Exosomes	High expression of miR-1290 is present in breast cancer (BC) patients in comparison to healthy subjects; miR-1290 is associated with lymph node metastasis and advanced stage of BC (TNM II/III vs I)	[234]
	Overexpression	Plasma and serum	Higher miR-1290 expression detected in BC patients than in healthy individuals; Expression of miR-1290 is higher in stages I-III than in stage IV and in HER-2 positive and triple-negative breast cancer (TNBC) subtypes	[235]

	Downregulation	Tissues; MCF-7 and T47D cell lines	Lower expression of miR-1290 in ER (high) Ki67 (low) BC is associated with tumor grade and correlated with better prognosis; miR-1290 targets *FOXA1* and *NAT1*; *NAT1* positively correlates with ER and progesterone expression, and is negatively correlated with tumor size and grade;	[236]
	Downregulation	Tissues	miR-1290 is downregulated in TNBC subtype; miR-1290 targets *FOXA1* and *BMP4;* miR-1290 signature is associated with poor prognosis	[238]
**Cervical cancer**	Overexpression	Serum	miR-1290 is upregulated in cervical cancer (CC) patients in comparison to healthy controls; miR-1290 expression increases along with CC stage	[133]
	Overexpression	HeLa-NGDR2, HeLa-R11 and Siha-R15 cell lines	miR-1290 is elevated in radioresistant cells; Expression of miR-1290 can be stimulated by radiation treatment that increases cells' survival	[243]
	Overexpression	CaSKi, HeLa and Siha cell lines; Tissues	miR-1290 is upregulated in cells with HPV infection upon 5′-aza-2′-deoxycytidine treatment; Upregulation of miR-1290 may be associated with DNA methylation status	[245]
**Endometrial cancer**	Overexpression	Plasma	miR-1290 is upregulated in plasma from high-grade endometrial cancer patients in comparison to controls	[134]
**Ovarian cancer**	Overexpression	HeyA8 and TYK-nu -derived exosomes; Serum	Level of miR-1290 is higher in exosomes collected from the culture media of HeyA8 and TYK-nu cells (high-grade serous ovarian carcinoma (HGSC) cells) than in immortalized ovarian surface epithelium cells; miR-1290 is upregulated in HGSC serum samples in comparison to healthy controls; miR-1290 can discriminate HGSC from malignancies of other histological types	[135]
	Overexpression	Ascitic fluid and ascites-derived lavage; Plasma	Increased miR-1290 in HGSC, mucinous and endometroid ovarian cancer (OC) especially at advanced stages; OC ascites-derived miR-1290 is overexpressed relative to the control plasma from the healthy postmenopausal women	[136]
	Overexpression	Tissues	miR-1290 is higher in recurrent serous OC than in the primary OC	[251]
	Overexpression	Tissues	Upregulation of miR-1290 in epithelial OC than in normal oviduct tissues	[252]
	Downregulation	Tissues; OVCAR and SKOV-3	Downregulation of miR-1290 by long-noncoding RNA CCAT1 promotes proliferation of OC cells	[253]
	Overexpression	Plasma	Elevated miR-1290 in presurgical OC plasma is a predictor of favorable OS; Level of miR-1290 decreases after surgery and chemotherapy, also in long overall survivors	[254]
**Prostate cancer**	Downregulation	Tissues	miR-1290 is slightly lower in prostate cancer (PCa) than in benign prostatic hyperplasia (BPH) and there is no difference between castrate-resistant prostate cancer (CRPC) and BPH	[256]
	Overexpression	Urinary extracellular vesicles	miR-1290 is significantly increased in PCa patients compared to BPH patients and it is associated with metastatic disease	[260]
	Overexpression	Plasma exosomes	miR-1290 is associated with poor OS in CRPC	[259]
	Overexpression	Serum	Five miRNAs coupled with miR-1290 (2-miRNA model) increase diagnostic value in early detection of PCa	[258]
**Oral cancer**	Overexpression	Tissues; Cal-27, SCC-9, SCC-25, and Tca-8113 cell lines	miR-1290 expression is higher in oral squamous cell carcinoma (OSCC) tissues than in normal tissues and in Cal-27, SCC-9, SCC-25, and Tca-8113 cell lines in comparison to normal human oral keratinocytes; High level of miR-1290 is associated with TNM stage, lymph node metastasis and shorter OS in OSCC patients; Upregulation of miR-1290 promotes migration, invasion and EMT *in vitro* by targeting *CCNG2*	[261]
	Downregulation	Plasma	Low level of miR-1290 in OSCC patients is related to clinical stage, differentiation degree, tumor diameter, lymph node metastasis, low PFS and OS	[137]
	Downregulation	Plasma; SAS and clinically relevant radioresistant cell lines (SAS-R)	Low miR-1290 in OSCC patients and in SAS cells in comparison to SAS-R cell line; The frequency of low-miR-1290 is higher in OSCC patients with low tumor	[138]

			differentiation, poor response to preoperative chemoradiotherapy, and it is also associated with shorter OS and DFS	
**Laryngeal cancer**	Overexpression	Tissues	miR-1290 is upregulated in supraglottic laryngeal squamous cell carcinoma (LaSCC) in comparison to matched normal mucosa and differentially expressed in supraglottic LaSCC with and without metastasis	[264]
	Overexpression	Tissues; LaSCC cell lines	miR-1290 targets 3'UTR of *MAF* mRNA; MAF protein distribution shows loss of MAF nuclear expression or complete absence in LaSCC samples in comparison to nuclear and cytoplasmic expression in normal mucosa	[267]
	Overexpression	Exosomes derived from AMC-HN-8 cell line	AMC-HN-8 cells are able to release miRNA-containing exosomes including miR-1290 in comparison to parental cells	[268]
**Cutaneous cancer**	Overexpression	Serum and tissues; cutaneous squamous cell carcinoma (CSCC) cell lines	miR-1290 is upregulated in tumor tissue and serum in organ transplant recipients with CSCC and in the supernatants of CSCC cell lines in comparison to cultured primary keratinocytes; Dysregulated miR-1290 in tissue is reflected in serum in patients without advanced disease and highly differentiated tumor; Circulating miR-1290 may serve as a promising tool in prediction, diagnosis and early detection of metastases in patients after excision of high-risk CSCC	[271]
**Brain cancer**	Overexpression	Tissues; LN-229 and U87 cell lines; Tumor mice model	miR-1290 is elevated in glioma samples compared to normal brain samples; miR-1290 targets *LHX6* and promotes proliferation and metastasis of cancer cells; Inhibition of *LHX6* expression promotes tumor growth *in vivo*	[272]
	Overexpression	U87 cell line	miR-1290 increases cell proliferation, migration, invasion and resistance to chemoradiation by targeting 3′ UTR of *SOCS4* mRNA; miR-1290 is a promising therapeutic target for glioblastoma therapy	[273]
	Overexpression	Tissues; LN229 and A172 cell lines; Tumor mice model	miR-1290 is overexpressed in glioma tissues compared to normal brain samples; miR-1290 targets 3'UTR of *FBXW7* mRNA; circBRAF (circular RNA) serves as a molecular sponge for miR-1290; Upregulation of circBRAF suppresses the development of glioma *in vitro* and *in vivo* by regulation of miR-1290/FBXW7 axis	[275]
	Overexpression	Tissues; T98G and LN18 cell lines; Tumor mice model	miR-1290 interacts with 3′ UTR of *RORA;* miR-1290 is a target of circRELN in glioma cells; Sevoflurane blocks the development of malignant glioma by upregulation of circRELN through circRELN-mediated miR-1290/RORA axis	[276]
	Overexpression	Tissues; Pediatric glioma stem cells (GSCs); Exosomes of GSCs	GSCs secrete exosomes rich in miR-1290 that alters expression of genes involved in tumorigenesis in recipient cells (normal neural stem cells, NSCs)	[277]
**Leukemia**	Overexpression	Tissues; SUP-B15 cells	Resveratrol targets miR-1290 and inhibits cell proliferation, migration, induces cell-cycle arrest and apoptosis in B-cell acute lymphoblastic leukemia (B-ALL); miR-1290 targets 3'UTR of *IGFBP3* which level is decreased in ALL patients	[278]
	Overexpression	Tissues	miR-1290 predicts shorter DFS in pediatric patients with B-ALL	[280]
**Conjunctival melanoma**	Downregulation	Tissues	miR-1290 is downregulated in primary conjunctival melanoma with subsequent metastatic spread in comparison to nonmetastatic tumors	[281]
